# ﻿Morphological and phylogenetic analysis reveal three new species *Phyllosticta* (Phyllostictaceae, Botryosphaeriales) in China

**DOI:** 10.3897/mycokeys.118.153609

**Published:** 2025-05-29

**Authors:** Meng-Yuan Zhang, Zhao-Xue Zhang, Du-Hua Li, Xiu-Guo Zhang, Ji-Wen Xia, Zhuang Li

**Affiliations:** 1 Shandong Provincial Key Laboratory for Biology of Vegetable Diseases and Insect Pests, College of Plant Protection, Shandong Agricultural University, Taian, 271018, China Shandong Agricultural University Taian China; 2 College of Agriculture and Forestry, Linyi University, Linyi, Shandong, 276000, China Linyi University Linyi China

**Keywords:** Ascomycota, morphology, new taxa, phylogeny, taxonomy

## Abstract

*Phyllosticta* (Phyllostictaceae, Botryosphaeriales) species have been reported worldwide and collected from various plant hosts. We proposed three new species, viz., *Phyllostictaelliptica***sp. nov.**, *P.rhododendri***sp. nov.**, and *P.wuzhishanensis***sp. nov.**, based on multi-locus phylogenetic analysis using a combined dataset of ITS rDNA, LSU, *tef1*, ACT, and GPDH, along with morphological characteristics. Moreover, *P.capitalensis*, isolated from the leaves of *Mangiferaindica*, is redescribed herein. We have re-examined the six species complexes: the *P.capitalensis* species complex (including 32 species), *P.concentrica* species complex (including 32 species), *P.cruenta* species complex (including 22 species), *P.owaniana* species complex (including six species), *P.rhodorae* species complex (including two species), and *P.vaccinii* species complex (including two species). Detailed descriptions and illustrations of the new species are provided. These findings enrich the biodiversity of fungi and provide reference for subsequent research.

## ﻿Introduction

*Phyllosticta* Pers. was established by Persoon in 1818. *Phyllostictaconvallariae* was designated as the type species (Donk 1968), which was later synonymized with *P.cruenta* (van der Aa 1973, Wikee et al. 2013a). *Phyllosticta* is a significant plant pathogenic genus known for causing leaf spots and fruit diseases on a wide range of economically important plants and ornamentals. Notable species include *P.citricarpa* (McAlpine) Aa, which causes citrus black spot (Baayen et al. 2002; Glienke et al. 2011); *P.ampelicida* (Engelm.) Aa, which is responsible for black rot disease on *Vitisvinifera* in North America (Kuo and Hoch 2018); and *Phyllostictaophiopogonis* Wulandari & K.D. Hyde, which causes leaf spots of *Ophiopogonjaponicus* in Thailand (Wikee et al. 2012). *Phyllostictacapitalensis* acts as a common endophyte with antagonistic potential against pathogenic species such as *P.citricarpa* and produces melanized appressoria for host penetration (Wikee et al. 2013b; Tran et al. 2019). A total of 3,226 *Phyllosticta* names are recorded in the Index Fungorum (accessed on 21 March 2025).

Since *Phyllosticta* is distinct from other genera in its family, Seaver (1922) initially classified it in the family Phyllostictaceae Fr. and placed it in the order Phyllostictales. However, subsequent studies (Crous et al. 2006; Schoch et al. 2006; Liu et al. 2012) reassigned *Phyllosticta* to the family Botryosphaeriaceae Theiss. & Syd., within the order Botryosphaeriales C.L. Schoch et al. However, phylogenetic analysis by Wikee et al. (2013a) positioned *Phyllosticta* in a clade sister to Botryosphaeriaceae, leading to its reclassification into the family Phyllostictaceae under the order Botryosphaeriales (Zhang et al. 2013). In recent years, an increasing number of new *Phyllosticta* species have been described based on a combination of molecular data and morphological characteristics (Su and Cai 2012; Wang et al. 2012, 2013; Wong et al. 2012; Zhang et al. 2012, 2013; Wikee et al. 2013a; Wulandari et al. 2013; Crous et al. 2014, 2016, 2017, 2018, 2019, 2021; Zhou et al. 2015; Guarnaccia et al. 2017; Lin et al. 2017; Hattori et al. 2020; Norphanphoun et al. 2020). Norphanphoun et al. (2020) compiled all species classified under *Phyllosticta* in GenBank, analyzing a comprehensive dataset of five loci, and consequently proposed six species complexes: the *P.capitalensis* species complex, *P.concentrica* species complex, *P.cruenta* species complex, *P.owaniana* species complex, *P.rhodorae* species complex, and *P.vaccinii* species complex. Subsequently, multiple novel taxa or new records were introduced based on morphological descriptions and multi-locus phylogenetic analysis (Zhang et al. 2023; Gomdola et al. 2024; Jiang et al. 2024).

The sexual morph of *Phyllosticta* species is characterized by erumpent, uniloculate, globose to subglobose ascomata with a central ostiole and pseudoparaphyses at maturity. Asci are clavate to ellipsoidal or ovoid, with an ocular chamber, while ascospores are aseptate, hyaline, ellipsoidal to limoniform, guttulate, and smooth-walled, often with mucoid caps at both ends (van der Aa 1973, Wong et al. 2012, Wikee et al. 2013a). The asexual morph produces conidia that are generally aseptate, hyaline, ovoid to ellipsoidal, and are surrounded by a mucilaginous sheath and possess an apical appendage. The morphology of appendages may vary depending on the growth medium (Wikee et al. 2013a). Spermatia are hyaline, aseptate, cylindrical to dumbbell-shaped, with guttules at each end. Because of overlapping morphological traits, species delimitation relies primarily on multi-locus phylogenetic analysis (Norphanphoun et al. 2020). Fungi associated with leaf spots were collected from *Mangiferaindica*, *Rhododendron×pulchrum*, and Rubusellipticusvar.obcordatus. We used sequences of five gene loci, including the internal transcribed spacer of ribosomal RNA (ITS rDNA), large subunit of ribosomal RNA (LSU rDNA), translation elongation factor 1 alpha (*tef1*), actin (ACT), and glycerol-3-phosphate dehydrogenase (GPDH). We also incorporated their morphology and then identified these fungi as three new species of the *P.concentrica* species complex.

## ﻿Materials and methods

### ﻿Isolation and morphological studies

Dead, healthy, and diseased leaves were collected from Hainan and Yunnan (from 2023 to 2024) and transported to the laboratory in paper bags. Fungal isolates were obtained using the tissue isolation method (Zhang et al. 2023, 2025). Leaf lesion fragments (5 × 5 mm) were excised from the margins of symptomatic tissues, surface-sterilized by sequential immersion in 75% ethanol for 30 seconds, rinsed in sterile distilled water, then treated with 5% sodium hypochlorite solution for 30 seconds, and finally rinsed in sterile distilled water for 1 minute (Jiang et al. 2021). After drying on sterilized tissue paper, the fragments were placed on 2% PDA and incubated at 25 °C for two to four days. Actively growing hyphal tips were subsequently transferred to fresh PDA plates and cultured for morphological examination. Colony morphology was documented on days 7 and 14 using a digital camera (Canon Powershot G7X, Canon Co., Ltd., Beijing, China). Micromorphological features were observed using an Olympus SZX10 stereomicroscope and Olympus BX53 microscope (Olympus Corporation, Tokyo, Japan), both equipped with an Olympus DP80 high-resolution color digital camera (Olympus Corporation, Tokyo, Japan) for imaging fungal structures. All fungal strains were stored in 10% sterilized glycerin at 4 °C for further studies. Structural measurements were performed using the Digimizer software (https://www.digimizer.com/), with thirty measurements taken for each morphological feature. Holotype specimens were deposited in the Herbarium of Plant Pathology, Shandong Agricultural University (**HSAUP**). Ex-type cultures and other living cultures were deposited in the China General Microbiological Culture Collection Center (CGMCC), Beijing, China, and the Shandong Agricultural University Culture Collection (SAUCC), Shandong, China. Taxonomic information for the newly described taxa was registered to MycoBank (http://www.mycobank.org).

### ﻿DNA extraction and sequencing

Fungal DNA was extracted from fungal mycelia grown on PDA using a modified cetyltrimethylammonium bromide (**CTAB**) protocol or a kit method (OGPLF-400, GeneOnBio Corporation, Changchun, China) for Sanger sequencing (Wang et al. 2023; Zhang et al. 2023, 2025). In this study, a total of eight pairs of primers were used; the internal transcribed spacer region (**ITS**) with intervening 5.8S rRNA gene, large subunit of rRNA gene (**LSU**), translation elongation factor 1-alpha gene (***tef1***), actin gene (**ACT**), and glyceraldehyde-3-phosphate dehydrogenase gene (**GPDH**) were amplified and sequenced using the primer pairs ITS5/ITS4 (White et al. 1990), LR0R/LR5 (White et al. 1990), EF1-728F/EF2 (O’Donnell et al. 1998; Carbone and Kohn 1999), ACT-512F/ACT-783R (Carbone and Kohn 1999), and GDF1/GAPDH (Myllys et al. 2002), respectively. Amplification reactions were performed in a 20 μL reaction volume, which contained 10 μL 2 × Hieff Canace® Plus PCR Master Mix (Shanghai, China) (with dye) (Yeasen Biotechnology, Cat. No. 10154ES03), 0.5 μL of each forward and reverse primer (10 μM) (TsingKe, Qingdao, China), and 1 μL template genomic DNA, adjusted with distilled deionized water to a total volume of 20 μL. PCR amplification products were visualized on 2% agarose electrophoresis gel. The Gel Extraction Kit (Cat: AE0101-C) (Shandong Sparkjade Biotechnology Co., Ltd.) was used for gel recovery. Sanger sequencing was performed using an Eppendorf Master Thermocycler (Hamburg, Germany) at the Youkang Biotechnology Co., Ltd. (Qingdao, China) bi-directionally. All sequences generated in this study were deposited in GenBank (Suppl. material 1).

### ﻿Phylogenetic analysis

The generated consensus sequences were subjected to BLAST searches in NCBI’s GenBank nucleotide database to identify closely related sequences (Zhang et al. 2000). For phylogenetic analysis based on ITS, LSU, *tef1*, ACT, and GPDH sequences, a subset of alignments from Jiang et al. (2024) was used as the backbone. Newly generated sequences from this study were aligned with related sequences retrieved from GenBank (Suppl. material 1) using the online tool MAFFT 7 with the Auto strategy (Katoh et al. 2019; http://mafft.cbrc.jp/alignment/server/) and corrected manually using BioEdit (Hall 2006). To determine the species identity of the isolates, phylogenetic analysis was initially performed for each locus separately, followed by a concatenated analysis of all loci (ITS-LSU-tef1-ACT-GPDH).

For phylogenetic analysis, we followed the methods by Zhang et al. (2023) using both maximum likelihood (ML) and Bayesian inference (BI) algorithms. ML was run on the CIPRES Science Gateway portal (Miller et al. 2012) (https://www.phylo.org/) or with RAxML-NG v1.2.1 (https://github.com/amkozlov/raxml-ng), and Bayesian inference (BI) analysis was performed using MrBayes v3.2.7a, running with 8 threads on a Linux system. For ML analysis, the default parameters were used, and 100 rapid bootstrap replicates were run with the GTR+G+I model of nucleotide evolution; BI analysis was performed using a fast bootstrap algorithm with an automatic stop option (Zhang et al. 2023). The burn-in fraction was set to 0.25, and posterior probabilities (PP) were determined from the remaining trees. All resulting trees were plotted using FigTree v. 1.4.4 (http://tree.bio.ed.ac.uk/software/figtree), and the layout of the trees was edited in Adobe Illustrator CC 2019.

## ﻿Results

### ﻿Phylogenetic analysis

**Figure 1.**
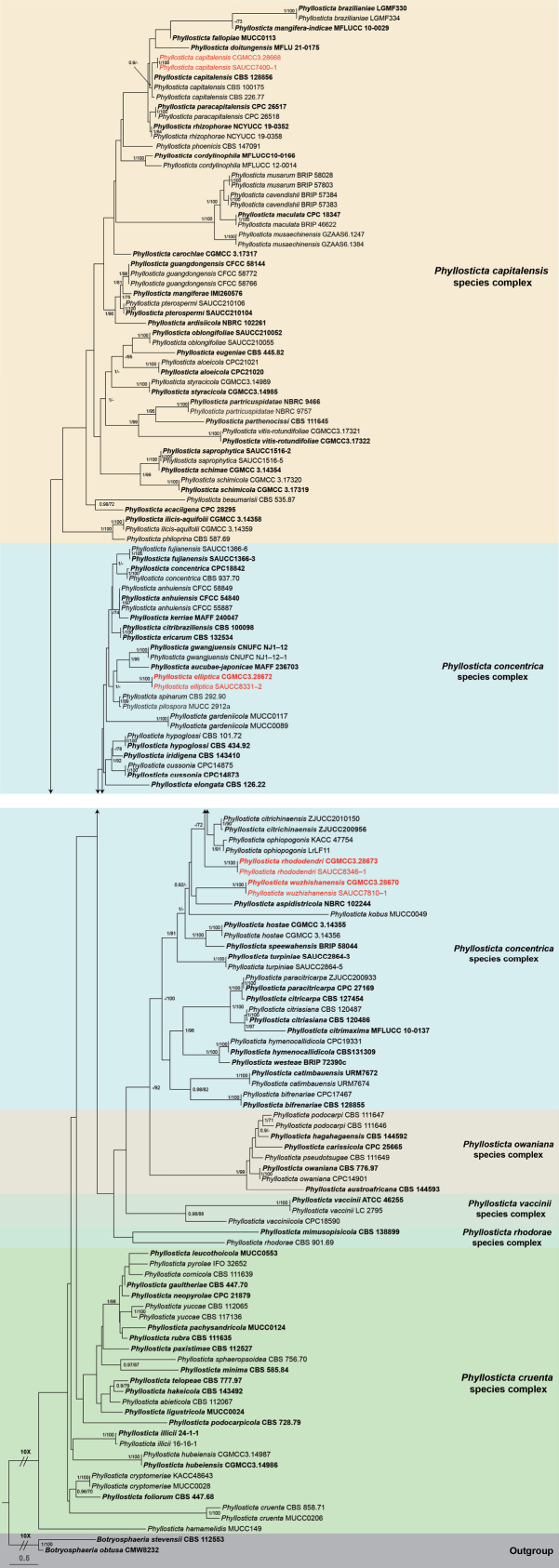
Phylogram of the *Phyllosticta*, inferred from a concatenated alignment of ITS, LSU, *tef1*, ACT, and GPDH sequences. *Botryosphaeriaobtusa* (CMW8232) and *B.stevensii* (CBS 112553) were used as outgroup taxa. BI posterior probabilities and ML bootstrap support values above 0.90 and 70% are shown at the first and second positions, respectively. Ex-type cultures are highlighted in bold, while strains obtained in this study are marked in red. Some branches have been shortened for layout optimization, indicated by double diagonal lines with the corresponding reduction factor. The scale bar at the bottom left represents the number of substitutions per site.

A total of 149 isolates representing *Phyllosticta* species were subjected to phylogenetic analysis, with *Botryosphaeriaobtusa* (CGMCC 3.14986) and *B.stevensii* (CBS 112553) designated as outgroup taxa. The final alignment consisted of 2,934 characters, corresponding to the following loci: 1–669 (ITS), 670–1,409 (LSU), 1,410–1,917 (*tef1*), 1,918–2,186 (ACT), and 2,187–2,934 (GPDH). Among these, 1,775 characters were constant, 215 were variable and parsimony-uninformative, and 944 were parsimony-informative. ML analysis yielded the best-scoring RAxML tree with a final likelihood value of –26,187.533107. The alignment contained 1,432 distinct patterns, with 36.76% undetermined characters or gaps. The estimated base frequencies were as follows: A = 0.200968, C = 0.314322, G = 0.270974, and T = 0.213736; substitution rates were AC = 1.162897, AG = 3.568637, AT = 1.418189, CG = 1.163532, CT = 6.776257, and GT = 1.0. The gamma distribution shape parameter alpha was estimated at 0.679976. As the ML and BI trees produced topologically congruent trees, only the ML tree (Fig. 1) is presented, with posterior probabilities and bootstrap provided for well-supported clades. Based on the five-gene phylogenetic framework (Fig. 1), the 149 isolates were assigned to 98 species. The present study identified three novel species, viz. *Phyllostictaelliptica* sp. nov., *P.rhododendri* sp. nov., and *P.wuzhishanensis* sp. nov.

## ﻿Taxonomy

### ﻿*Phyllostictacapitalensis* species complex

Based on molecular analysis and morphological characteristics, the *Phyllostictacapitalensis* species complex comprises 32 species: *P.acaciigena*, *P.aloeicola*, *P.ardisiicola*, *P.beaumarisii*, *P.brazilianiae*, *P.capitalensis*, *P.carochlae*, *P.cavendishii*, *P.cordylinophila*, *P.doitungensis*, *P.eugeniae*, *P.fallopiae*, *P.guangdongensis*, *P.ilicis-aquifolii*, *P.maculata*, *P.mangiferae*, *P.mangifera-indicae*, *P.musaechinensis*, *P.musarum*, *P.oblongifoliae*, *P.paracapitalensis*, *P.parthenocissi*, *P.partricuspidatae*, *P.philoprina*, *P.phoenicis*, *P.pterospermi*, *P.rhizophorae*, *P.saprophytica*, *P.schimae*, *P.schimicola*, *P.styracicola*, and *P.vitis-rotundifoliae*.

#### ﻿ Phyllosticta capitalensis

Taxon classificationFungiBotryosphaerialesPhyllostictaceae

Henn., Hedwigia 48: 13 (1908)

51CE15EB-41BF-5200-B9E9-83172F695A7A

Fig. 2

##### Description.

Leaf endogenic and associated with leaves of *Mangiferaindica*. Sexual morph: Not observed. Asexual morph: ***Conidiomata*** pycnidial, mostly aggregated in clusters, black, erumpent. In PDA culture, exuding colorless to opaque conidial masses within 12 d or longer. ***Pycnidial walls*** multilayered, ***textura angularis***, brown, inner walls of hyaline. ***Conidiophores*** indistinct, often reduced to conidiogenous cells. ***Conidiogenous cells*** 6–12 × 2.2–2.8 μm, subcylindrical, ampulliform, hyaline, smooth. ***Conidia*** 6.7–12 × 3.6–5.8 μm (L/W 1.42–1.53), ovoid, ampulliform, ellipsoidal to subglobose, hyaline, aseptate, thin and smooth walled, multi-guttulate, or with a single large central guttule, surrounded by a mucilaginous sheath. ***Sheath*** 2.6–2.9 μm thick, thicker on both sides, and bearing a hyaline, apical mucoid appendage. ***Appendages*** 5.7–6.7 × 1.2–2.1 μm, flexible, unbranched, tapering towards an acutely rounded tip.

**Figure 2.**
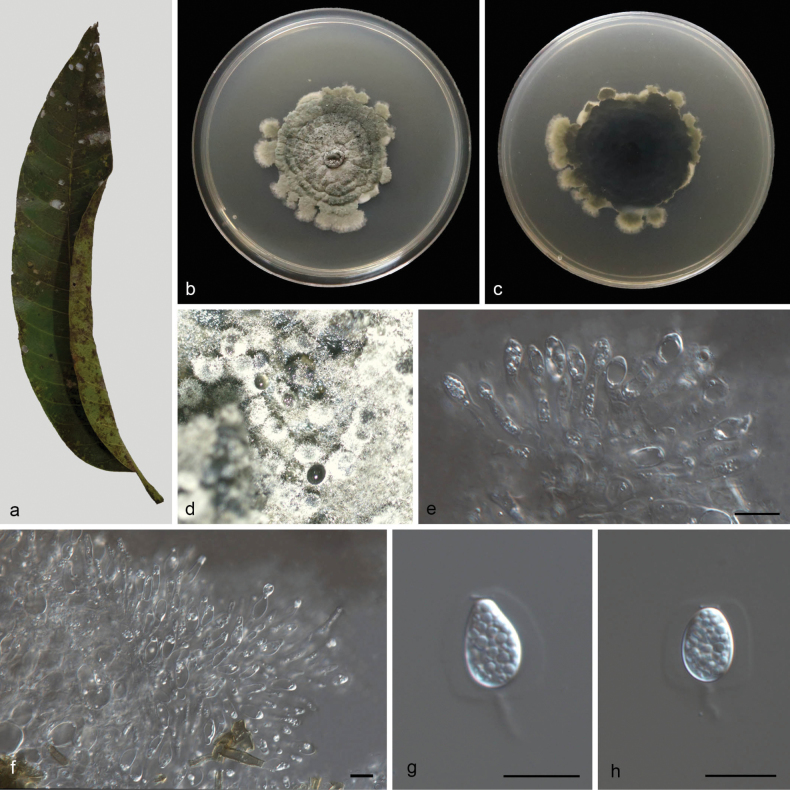
*Phyllostictacapitalensis* (CGMCC3.28668) **a** diseased leaf of *Mangiferaindica***b, c** colonies (left-above, right-reverse) after 14 d on PDA **d** conidiomata **e, f** conidiogenous cells with conidia **g, h** conidia. Scale bars: 10 μm (**e–h**).

##### Culture characteristics.

Colonies on PDA 46–52 mm in diameter after 14 d at 25 °C in darkness, with a growth rate of 3.3–3.7 mm/day, undulate at edge, grey white to greenish-black on obverse and reverse.

##### Additional specimen examined.

China • Hainan Province, Wanning City, Xinglong tropical botanical garden, on diseased leaves of *Mangiferaindica* L., 28 March 2024, M.Y. Zhang (HSAUP7403), living culture CGMCC3.28668; *ibid*., Z.X. Zhang (HSAUP7400), living culture SAUCC7400–1.

##### Notes.

The holotype (CBS 128856) of *P.capitalensis* was collected from *Stanhopeagraveolens* (Glienke et al. 2011). Two isolates (CGMCC3.28668 and SAUCC7400–1), collected from diseased leaves of *Mangiferaindica*, cluster in the *P.capitalensis* clade (Fig. 1). In morphology, they possess the same morphological characters, such as subcylindrical to ampullate conidiogenous cells (6–12 × 2.2–2.8 vs. 7–10 × 3–5 μm), ellipsoidal to subglobose conidia (6.7–12 × 3.6–5.8 vs. 11–12 × 6–7 μm), and hyaline, apical mucoid appendages (5.7–6.7 × 1.2–2.1 vs. 6–8 × 1–1.5 μm). Therefore, we defined these two isolates as *P.capitalensis*.

### ﻿*Phyllostictaconcentrica* species complex

Based on molecular analysis and morphological characteristics, the *Phyllostictaconcentrica* species complex comprises 32 species: *P.anhuiensis*, *P.aspidistricola*, *P.aucubae-japonicae*, *P.bifrenariae*, *P.catimbauensis*, *P.citriasiana*, *P.citribraziliensis*, *P.citricarpa*, *P.citrichinensis*, *P.citri-maxima*, *P.concentrica*, *P.cussonia*, *P.elliptica*, *P.elongata*, *P.ericarum*, *P.fujianensis*, *P.gardeniicola*, *P.gwangjuensis*, *P.hostae*, *P.hymenocallidicola*, *P.hypoglossi*, *P.iridigena*, *P.kerriae*, *P.kobus*, *P.ophiopogonis*, *P.paracitricarpa*, *P.pilospora*, *P.rhododendri*, *P.speewahensis*, *P.turpiniae*, *P.westeae*, and *P.wuzhishanensis*.

#### ﻿ Phyllosticta elliptica

Taxon classificationFungiBotryosphaerialesPhyllostictaceae

M.Y. Zhang, Z.X. Zhang & X.G. Zhang sp. nov.

B28C49F7-70A1-559A-B228-9EEE7D319D08

857335

Fig. 3

##### Etymology.

The specific epithet “*elliptica*” refers to the genus name of the host plant Rubusellipticusvar.obcordatus.

##### Type.

China • Yunnan Province, Hongta District, Yuxi City, Longma Mountain Scenic Area, on diseased leaves of Rubusellipticusvar.obcordatus (Franch.) Focke, 12 May 2024, M.Y. Zhang (holotype HSAUP8332), ex-type living culture CGMCC3.28672.

##### Description.

Leaf endogenic and associated with leaves of Rubusellipticusvar.obcordatus. Sexual morph: Not observed. Asexual morph: ***Conidiomata*** pycnidial, mostly aggregated in clusters, black, erumpent. In PDA culture, exuding white conidial masses within 15 days or longer. ***Pycnidial walls*** multilayered, ***textura angularis***, brown, inner walls of hyaline. ***Conidiophores*** indistinct, often reduced to conidiogenous cells. ***Conidiogenous cells*** 7–15 × 1.8–4 μm, cylindrical, hyaline, smooth. ***Conidia*** 12–15 × 7.5–11 μm (L/W 1.17–1.54), ovoid, ampulliform, ellipsoidal to subglobose, hyaline, aseptate, thin and smooth walled, multi-guttulate, or with a single large central guttule, surrounded by a mucilaginous sheath. ***Sheath*** 1.3–1.8 μm thick, thicker on both sides, and bearing a hyaline, apical mucoid appendage. ***Appendages*** 4–12 × 1–1.2 μm, flexible, unbranched, tapering towards an acutely rounded tip.

**Figure 3.**
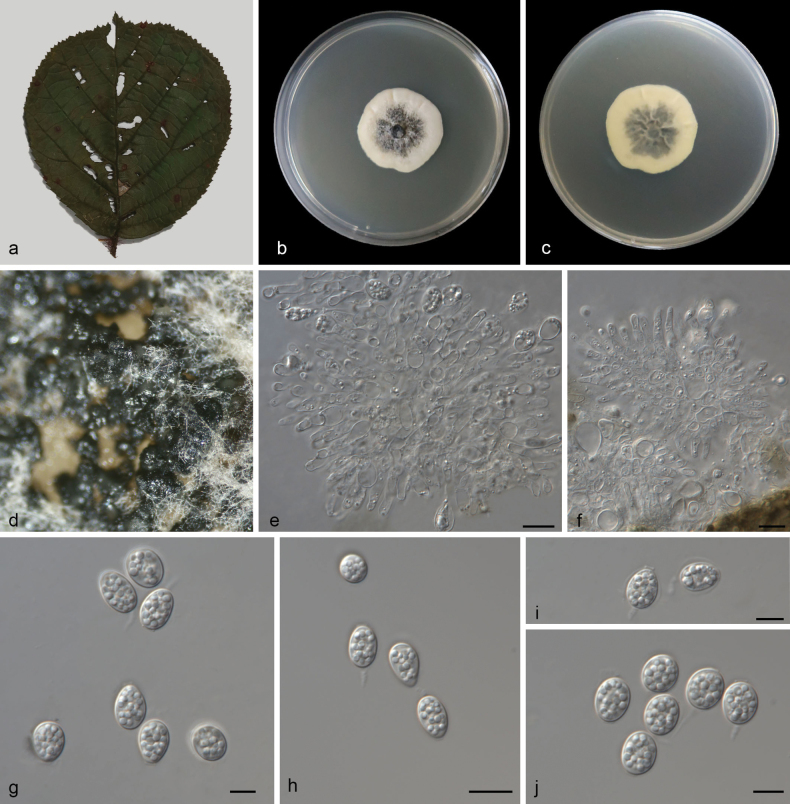
*Phyllostictaelliptica* (CGMCC3.28672) **a** diseased leaf of Rubusellipticusvar.obcordatus**b, c** colonies (left-above, right-reverse) after 14 d on PDA **d** conidiomata **e, f** conidiogenous cells with conidia **g–j** conidia. Scale bars: 10 μm (**e–j**).

##### Culture characteristics.

Colonies on PDA 33–36 mm in diameter after 14 d at 25 °C in darkness, with a growth rate of 2.3–2.6 mm/day, undulate at edge, creamy white to black in obverse and reverse.

##### Additional specimen examined.

China • Yunnan Province, Hongta District, Yuxi City, Longma Mountain Scenic Area, on dead leaves, 12 May 2024, M.Y. Zhang (HSAUP8331), living culture SAUCC8331–2.

##### Notes.

*Phyllostictaelliptica* is closely related to *P.aucubae-japonicae* (MAFF 236703) and *P.gwangjuensis* (CNUFC NJ1–12 and CNUFC NJ1-12-1) based on DNA sequence data in BLAST searches and phylogenetic analysis (Fig. 1). However, *P.elliptica* differs from *P.aucubae-japonicae* by 70 nucleotides (31/628 in ITS, 0/737 in LSU, 22/266 in *tef*, 17/239 in ACT, and 0/725 in GPDH) and from *P.gwangjuensis* by 60 nucleotides (25/634 in ITS, 0/737 in LSU, 16/376 in *tef1*, 19/214 in ACT, and 0/725 in GPDH). In morphology, they are distinguished by different hosts (Rubusellipticusvar.obcordatus vs. *Aucubajaponica* vs. *Torreyanucifera*) and longer conidia in *Phyllostictaelliptica* than in *P.aucubae-japonicae* and *P.gwangjuensis* (12–15 × 7.5–11 μm (L/W 1.17–1.54) vs. 10–13 × 5–8.5 μm (L/W 1.41–1.65) vs. (8.5–)10–13.5 × 7–9(–9.5) μm (L/W 1.40–1.53)) (Hernandez-Restrepo et al. 2016; Nguyen et al. 2022). Therefore, based on morphology and phylogenetic evidence, we establish this fungus as *Phyllostictaelliptica* sp. nov.

#### ﻿ Phyllosticta rhododendri

Taxon classificationFungiBotryosphaerialesPhyllostictaceae

M.Y. Zhang, Z.X. Zhang & X.G. Zhang sp. nov.

A7908DF2-AEAB-5FA7-821D-8A0588E45590

857221

Fig. 4

##### Etymology.

The specific epithet “*rhododendri*” refers to the host plant Rhododendron×pulchrum Sweet.

##### Type.

China • Yunnan Province, Hongta District, Yuxi City, Longma Mountain Scenic Area, on diseased leaves of Rhododendron×pulchrum, 13 May 2024, M.Y. Zhang (holotype HSAUP8342), ex-type living culture CGMCC3.28673.

##### Description.

Leaf endogenic and associated with leaves of Rhododendron×pulchrum. Sexual morph: Not observed. Asexual morph: ***Conidiomata*** pycnidial, mostly aggregated in clusters, black, erumpent. In PDA culture, exuding white conidial masses within 10 days or longer. ***Pycnidial wall*** multi-layered, ***textura angularis***, brown to dark brown. ***Conidiophores*** indistinct, often reduced to conidiogenous cells. ***Conidiogenous cells*** 7–16 × 1.4–5.2 μm, subcylindrical, ampulliform, hyaline, smooth. ***Conidia*** 8.5–11.5 × 6.8–9.4 µm (L/W 1.33–1.53), ovoid, ampulliform, ellipsoidal to subglobose, hyaline, aseptate, thin and smooth walled, multi-guttulate, or with a single large central guttule, surrounded by a mucilaginous sheath. ***Sheath*** 0.8–1.5 μm thick, thicker on both sides, and bearing a hyaline, apical mucoid appendage. ***Appendages*** 3–7.5 × 1–1.2 μm, flexible, unbranched, tapering towards an acutely rounded tip. Spermatia 6.3–8.2 × 1–1.5 μm, occurring in conidioma with conidia, hyaline, smooth, guttulate to granular, bacilliform.

**Figure 4.**
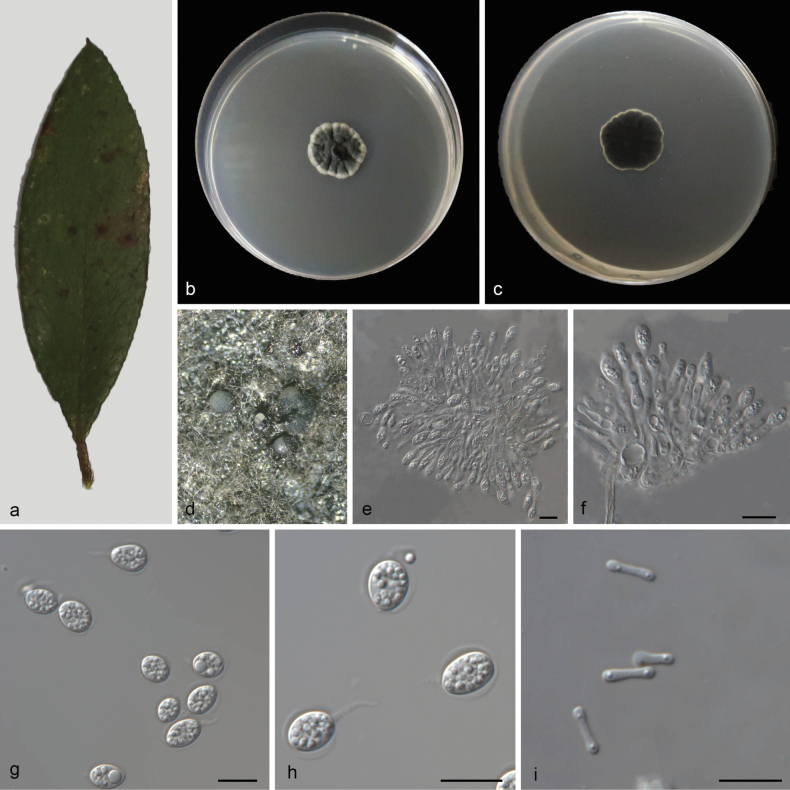
*Phyllostictarhododendri* (CGMCC3.28673) **a** diseased leaf of *Rhododendron×pulchrum***b, c** colonies (left-above, right-reverse) after 14 days on PDA **d** conidiomata **e, f** conidiogenous cells with conidia **g, h** conidia **i** spermatia. Scale bars: 10 μm (**e–i**).

##### Culture characteristics.

Colonies on PDA 19–22 mm in diameter after 14 days at 25 °C in darkness, with a growth rate of 1.4–1.6 mm/day, greenish black with white edge on obverse and reverse.

##### Additional specimens examined.

China • Yunnan Province, Hongta District, Yuxi City, Longma Mountain Scenic Area, on diseased leaves of Rhododendron×pulchrum, 13 May 2024, Z.X. Zhang (HSAUP8346), living culture SAUCC8346–1.

##### Notes.

Phylogenetic analysis showed that *Phyllostictarhododendri* formed an independent clade that is sister to a large clade comprising over ten species (*P.anhuiensis*, *P.aucubae-japonicae*, *P.citribraziliensis*, *P.citrichinaensis*, *P.concentrica*, *P.cussonia*, *P.elongata*, *P.elliptica*, *P.ericarum*, *P.fujianensis*, *P.gardeniicola*, *P.gwangjuensis*, *P.hypoglossi*, *P.iridigena*, *P.kerriae*, *P.ophiopogonis*, and *P.spinarum*) (Fig. 1). In morphology, *Phyllostictarhododendri* was found to produce spermatia, a structure that is rarely observed within this genus. Therefore, we establish this fungus as *Phyllostictaelliptica* sp. nov.

#### ﻿ Phyllosticta wuzhishanensis

Taxon classificationFungiBotryosphaerialesPhyllostictaceae

M.Y. Zhang, Z.X. Zhang & X.G. Zhang sp. nov.

371DA4D1-75A3-528F-9C51-EED858886A6C

857226

Fig. 5

##### Etymology.

The epithet “*wuzhishanensis*” pertains to Wuzhishan National Nature Reserve, where the type was collected.

##### Type.

China • Hainan Province, Wuzhishan National Nature Reserve, on saprophytic (dead leaves) leaves, 28 March 2024, M.Y. Zhang (holotype HSAUP7814), ex-type living culture CGMCC3.28670.

##### Description.

***Endophytic*** on saprophytic (dead leaves) leaves. Sexual morph: Not observed. Asexual morph: ***Conidiomata*** pycnidial, mostly aggregated in clusters, black, erumpent. In PDA culture, exuding colorless to opaque conidial masses within 12 days or longer. Pycnidial walls multilayered, ***textura angularis***, brown, inner walls of hyaline. Conidiophores indistinct, often reduced to conidiogenous cells. ***Conidiogenous cells*** 6.5–14.5 × 3–4 μm, subcylindrical, ampulliform, hyaline, smooth. ***Conidia*** 5.5–10 × 3.8–8.6 μm (L/W 1.27–1.83), ovoid, ampulliform, ellipsoidal to subglobose, hyaline, aseptate, thin and smooth walled, multi-guttulate, or with a single large central guttule, surrounded by a mucilaginous sheath. ***Sheath*** 1.6–2 μm thick, thicker on both sides, and bearing a hyaline, apical mucoid appendage. ***Appendages*** 10–13 × 1.2–1.5 μm, flexible, unbranched, tapering towards an acutely rounded tip. ***Spermatia*** 6.3–8.2 × 1–1.5 μm, occurring in conidioma with conidia, hyaline, smooth, guttulate to granular, bacilliform.

**Figure 5.**
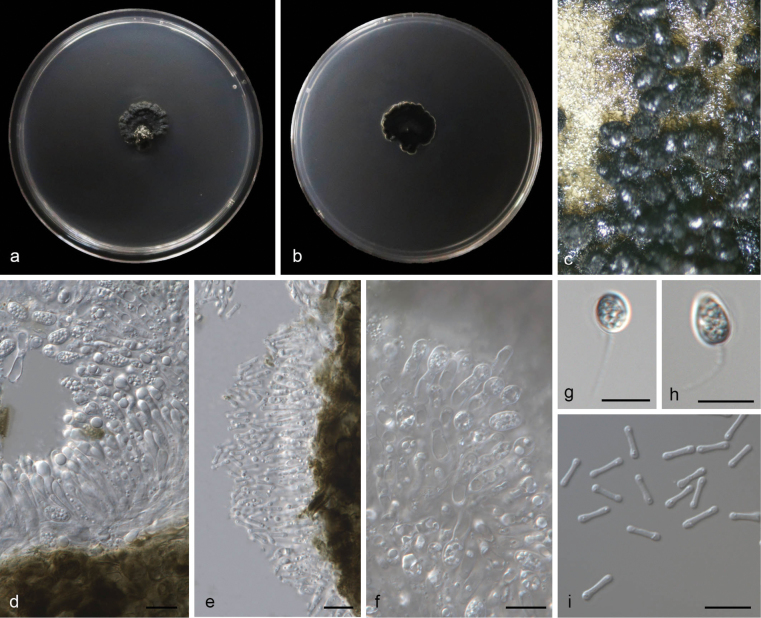
*Phyllostictawuzhishanensis* (CGMCC3.28670) **a, b** colonies (left-above, right-reverse) after 14 d on PDA **c** conidiomata **d–f** conidiogenous cells with conidia **g, h** conidia **i** spermatia. Scale bars: 10 μm (**d–i**).

##### Culture characteristics.

Colonies on PDA 17–21 mm in diameter after 14 days at 25 °C in darkness, with a growth rate of 1.2–1.5 mm/day, undulate at edge, black on obverse and reverse.

##### Additional specimen examined.

China • Hainan Province: Wuzhishan National Nature Reserve, on saprophytic (dead leaves) leaves, 28 March 2024, M.Y. Zhang (HSAUP7810), living culture SAUCC7810–1.

##### Notes.

*Phyllostictawuzhishanensis* is closely related to *P.aspidistricola* (NBRC 102244) based on DNA sequence data in BLAST searches and phylogenetic analysis (Fig. 1). However, *P.wuzhishanensis* differs from *P.aspidistricola* by 69 nucleotides (40/621 in ITS, 0/737 in LSU, 0/411 in *tef1*, 29/252 in ACT, and 0/727 GPDH). In morphology, they are distinguished by different hosts (dead leaves vs. *Aspidistraelatior*) and conidial size (5.5–10 × 3.8–8.6 μm (L/W 1.27–1.83) in *P.wuzhishanensis* vs. 9.5–12.5 × 8.5–10 μm (L/W 1.43–1.63) in *P.aspidistricola*) (Motohashi et al. 2008). Based on morpho-molecular evidence, we establish this fungus as *Phyllostictawuzhishanensis* sp. nov.

### ﻿*Phyllostictacruenta* species complex

Based on molecular analysis and morphological characteristics, the *Phyllostictacruenta* species complex comprises 22 species: *P.abieticola*, *P.cornicola*, *P.cruenta*, *P.cryptomeriae*, *P.foliorum*, *P.gaultheriae*, *P.hakeicola*, *P.hamamelidis*, *P.hubeiensis*, *P.illicii*, *P.leucothoicola*, *P.ligustricola*, *P.minima*, *P.neopyrolae*, *P.pachysandricola*, *P.paxistimae*, *P.podocarpicola*, *P.pyrolae*, *P.rubra*, *P.sphaeropsoidea*, *P.telopeae*, and *P.yuccae*.

### ﻿*Phyllostictaowaniana* species complex

Based on molecular analysis and morphological characteristics, the *Phyllostictaowaniana* species complex comprises 6 species: *P.austroafricana*, *P.carissicola*, *P.hagahagaensis*, *P.owaniana*, *P.podocarpi*, and *P.pseudotsugae*.

### ﻿*Phyllostictarhodorae* species complex

Based on molecular analysis and morphological characteristics, the *Phyllostictarhodorae* species complex comprises 2 species: *P.mimusopisicola* and *P.rhodorae*.

### ﻿*Phyllostictavaccinii* species complex

Based on molecular analysis and morphological characteristics, the *Phyllostictavaccinii* species complex comprises 2 species: *P.vaccinii* and *P.vacciniicola*.

## ﻿Discussion

In modern fungal taxonomy, integrating molecular data with morphological characteristics has become essential. As the limitations of traditional classification systems become increasingly evident, mycologists have turned to divergence time estimates and phylogenomic data to define clearer taxonomic boundaries (Baayen et al. 2002; Glienke et al. 2011; Wikee et al. 2013a, 2013b; Crous et al. 2014, 2016, 2017, 2018, 2019, 2021; Zhou et al. 2015; Guarnaccia et al. 2017; Lin et al. 2017; Hattori et al. 2020; Norphanphoun et al. 2020; Zhang et al. 2023, 2025). Historically, the identification of *Phyllosticta* species has relied on morphological characteristics and host associations. However, due to significant overlap in morphological traits among species, recognizing homologous characters has remained a challenge, leading to long-standing difficulties in *Phyllosticta* species delimitation (Norphanphoun et al. 2020). The advent of molecular phylogenetic approaches has greatly enhanced species recognition and the classification of species complexes (Baayen et al. 2002; Okane et al. 2003; Motohashi et al. 2009; Wulandari et al. 2009; Glienke et al. 2011; Wikee et al. 2012). The ITS region is widely used as a primary genetic marker for genus-level identification (White et al. 1990). However, for accurate species delimitation, additional loci such as LSU, *tef1*, ACT, and GPDH are required to achieve sufficient resolution (Norphanphoun et al. 2020). To date, six major species complexes encompassing 96 accepted species have been recognized within *Phyllosticta*, including *P.capitalensis* species complex (including 32 species), *P.concentrica* species complex (including 32 species), *P.cruenta* species complex (including 22 species), *P.owaniana* species complex (including six species), *P.rhodorae* species complex (including two species), and *P.vaccinii* species complex (including two species).

In this study, we described and illustrated four *Phyllosticta* isolates recovered from three host genera (*Mangiferaindica*, *Rhododendron×pulchrum*, and Rubusellipticusvar.obcordatus) based on a multi-locus phylogenetic analysis combined with morphological observations in culture. Three new species were proposed, including *Phyllostictaelliptica* sp. nov. (Rubusellipticusvar.obcordatus), *P.rhododendri* sp. nov. (*Rhododendron×pulchrum*), and *P.wuzhishanensis* sp. nov. (saprophytic leaves). These species were assigned to the *P.concentrica* species complex. Additionally, *Phyllostictapilospora* (MUCC 2912a) was confirmed as a member of the *P.concentrica* species complex (Hattori et al. 2020). Phylogenetic analysis (Fig. 1, Suppl. material 1) and morphological comparisons revealed that *P.pilospora* (MUCC 2912a) and *P.spinarum* (CBS 292.90) are closely related (Hattori et al. 2020; Gomzhina and Gannibal 2023).

The USDA Fungal Database contains over 7,500 records of *Phyllosticta* species associated with plant hosts (excluding synonyms) (https://fungi.ars.usda.gov/, accessed 21 March 2025) (Okane et al. 2001, 2003; Baayen et al. 2002; Glienke et al. 2011; Wikee et al. 2013b; Wu et al. 2014; Zhang et al. 2015; Tran et al. 2019; Hattori et al. 2020; Norphanphoun et al. 2020; Zhang et al. 2022; Gomdola et al. 2024; Jiang et al. 2024). Additionally, *Phyllostictacapitalensis* is recognized as a globally distributed endophyte and weak plant pathogen, with over 400 host-associated records listed in the USDA Fungal Database (Glienke-Blanco et al. 2002; Silva and Pereira 2007; Silva et al. 2008; Wikee et al. 2013a). These findings suggest that certain *Phyllosticta* taxa exhibit widespread endophytic lifestyles, warranting further investigation into their ecological roles and evolutionary adaptations.

## Supplementary Material

XML Treatment for Phyllosticta capitalensis

XML Treatment for Phyllosticta elliptica

XML Treatment for Phyllosticta rhododendri

XML Treatment for Phyllosticta wuzhishanensis

## ﻿Additional information

### Author ORCIDs

Meng-Yuan Zhang https://orcid.org/0009-0002-3068-7491

Zhao-Xue Zhang https://orcid.org/0000-0002-4824-9716

Du-Hua Li https://orcid.org/0009-0006-5200-2034

Xiu-Guo Zhang https://orcid.org/0000-0001-9733-8494

Ji-Wen Xia https://orcid.org/0000-0002-7436-7249

### Data availability

All of the data that support the findings of this study are available in the main text or Supplementary Information.

## Supplementary materials

Supplementary material 1 Species and GenBank accession numbers of DNA sequences used in this study Data type docx https://binary.pensoft.net/file/1341124This dataset is made available under the Open Database License (http://opendatacommons.org/licenses/odbl/1.0/). The Open Database License (ODbL) is a license agreement intended to allow users to freely share, modify, and use this Dataset while maintaining this same freedom for others, provided that the original source and author(s) are credited.Meng-Yuan Zhang, Zhao-Xue Zhang, Du-Hua Li, Xiu-Guo Zhang, Ji-Wen Xia, Zhuang Li

Supplementary material 2 The sequence of phylogenetic analysis Data type txt https://binary.pensoft.net/file/1341125This dataset is made available under the Open Database License (http://opendatacommons.org/licenses/odbl/1.0/). The Open Database License (ODbL) is a license agreement intended to allow users to freely share, modify, and use this Dataset while maintaining this same freedom for others, provided that the original source and author(s) are credited.Meng-Yuan Zhang, Zhao-Xue Zhang, Du-Hua Li, Xiu-Guo Zhang, Ji-Wen Xia, Zhuang Li

Supplementary material 3 Single phylogenetic analysis Data type pdf https://binary.pensoft.net/file/1341126This dataset is made available under the Open Database License (http://opendatacommons.org/licenses/odbl/1.0/). The Open Database License (ODbL) is a license agreement intended to allow users to freely share, modify, and use this Dataset while maintaining this same freedom for others, provided that the original source and author(s) are credited.Meng-Yuan Zhang, Zhao-Xue Zhang, Du-Hua Li, Xiu-Guo Zhang, Ji-Wen Xia, Zhuang Li
