# Supplementary material for: ﻿Morphological and phylogenetic analysis reveal three new species Phyllosticta (Phyllostictaceae, Botryosphaeriales) in China
Source: MycoKeys. 2025 May 29;118:35–54. doi: 10.3897/mycokeys.118.153609 (PMC12142213; doi:10.3897/mycokeys.118.153609)
Supplement: Supplementary material 1 — Species and GenBank accession numbers of DNA sequences used in this study [file mycokeys-118-035-s001.docx]

Table 1. Species and GenBank accession numbers of DNA sequences used in this study.

| Species | Voucher2 | Host/Substrate | Country | GenBank accession number | | | | |
| --- | --- | --- | --- | --- | --- | --- | --- | --- |
|  |  |  |  | ITS | LSU | tef1 | ACT | gapdh |
| ***Phyllosticta capitalensis* species complex** | | | | | | | | |
| *P. acaciigena* | CPC 28295 ^T^ | *Acacia suaveolens* | Australia | [KY173433](http://www.ncbi.nlm.nih.gov/nuccore/KY173433) | [KY173523](http://www.ncbi.nlm.nih.gov/nuccore/KY173523) | NA | [KY173570](http://www.ncbi.nlm.nih.gov/nuccore/KY173570) | NA |
| *P. aloeicola* | CPC 21020 ^T^ | *Aloe ferox* | South Africa | [KF154280](http://www.ncbi.nlm.nih.gov/nuccore/KF154280) | [KF206214](http://www.ncbi.nlm.nih.gov/nuccore/KF206214) | [KF289193](http://www.ncbi.nlm.nih.gov/nuccore/KF289193) | [KF289311](http://www.ncbi.nlm.nih.gov/nuccore/KF289311) | [KF289124](http://www.ncbi.nlm.nih.gov/nuccore/KF289124) |
|  | CPC 21021 | *Aloe ferox* | South Africa | [KF154281](http://www.ncbi.nlm.nih.gov/nuccore/KF154281) | [KF206213](http://www.ncbi.nlm.nih.gov/nuccore/KF206213) | [KF289194](http://www.ncbi.nlm.nih.gov/nuccore/KF289194) | [KF289312](http://www.ncbi.nlm.nih.gov/nuccore/KF289312) | [KF289125](http://www.ncbi.nlm.nih.gov/nuccore/KF289125) |
| *P. ardisiicola* | NBRC 102261 ^T^ | *Ardisia crenata* | Japan | [AB454274](http://www.ncbi.nlm.nih.gov/nuccore/AB454274) | NA | NA | [AB704216](http://www.ncbi.nlm.nih.gov/nuccore/AB704216) | NA |
| *P. beaumarisii* | CBS 535.87 | *Muehlenbekia adpressa* | Australia | [NR_145235](http://www.ncbi.nlm.nih.gov/nuccore/NR_145235) | [NG_058040](http://www.ncbi.nlm.nih.gov/nuccore/NG_058040) | [KF766429](http://www.ncbi.nlm.nih.gov/nuccore/KF766429) | [KF306232](http://www.ncbi.nlm.nih.gov/nuccore/KF306232) | [KF289074](http://www.ncbi.nlm.nih.gov/nuccore/KF289074) |
| *P. brazilianiae* | LGMF 330 ^T^ | *Mangifera indica* | Brazil | [JF343572](http://www.ncbi.nlm.nih.gov/nuccore/JF343572) | [KF206217](http://www.ncbi.nlm.nih.gov/nuccore/KF206217) | [JF343593](http://www.ncbi.nlm.nih.gov/nuccore/JF343593) | [JF343656](http://www.ncbi.nlm.nih.gov/nuccore/JF343656) | [JF343758](http://www.ncbi.nlm.nih.gov/nuccore/JF343758) |
|  | LGMF 334 | *Mangifera indica* | Brazil | [JF343566](http://www.ncbi.nlm.nih.gov/nuccore/JF343566) | [KF206215](http://www.ncbi.nlm.nih.gov/nuccore/KF206215) | [JF343587](http://www.ncbi.nlm.nih.gov/nuccore/JF343587) | [JF343650](http://www.ncbi.nlm.nih.gov/nuccore/JF343650) | [JF343752](http://www.ncbi.nlm.nih.gov/nuccore/JF343752) |
| *P. capitalensis* | CBS 128856 ^T^ | *Stanhopea* sp*.* | Brazil | [JF261465](http://www.ncbi.nlm.nih.gov/nuccore/JF261465) | [KF206304](http://www.ncbi.nlm.nih.gov/nuccore/KF206304) | [JF261507](http://www.ncbi.nlm.nih.gov/nuccore/JF261507) | [JF343647](http://www.ncbi.nlm.nih.gov/nuccore/JF343647) | [JF343776](http://www.ncbi.nlm.nih.gov/nuccore/JF343776) |
|  | CBS 226.77 | *Baccaurea ramiflora* | Brazil | FJ538336 | KF206289 | FJ538394 | FJ538452 | JF343718 |
|  | CBS 356.52 | *Paphiopedilum callosum* | Germany | FJ538342 | KF206300 | FJ538400 | FJ538458 | KF289087 |
|  | CBS 100175 | *Ilex* sp. | Not given | FJ538320 | KF206327 | FJ538378 | FJ538436 | JF343699 |
|  | **CGMCC3.28668** | ***Mangifera indica*** | **China** | **PQ891862** | **PQ891854** | **PQ885498** | **PQ885490** | **PQ885506** |
|  | **SAUCC7400-1** | ***Mangifera indica*** | **China** | **PQ891863** | **PQ891855** | **PQ885499** | **PQ885491** | **PQ885507** |
| *P. carochlae* | CGMCC3.17317 ^T^ | *Caryota ochlandra* | China | [KJ847422](http://www.ncbi.nlm.nih.gov/nuccore/KJ847422) | NA | [KF289178](http://www.ncbi.nlm.nih.gov/nuccore/KF289178) | [KF289273](http://www.ncbi.nlm.nih.gov/nuccore/KF289273) | [KF289092](http://www.ncbi.nlm.nih.gov/nuccore/KF289092) |
| *P. cavendishii* | BRIP 57384 | *Musa* cv. *Lady finger* | Australia | [KC117644](http://www.ncbi.nlm.nih.gov/nuccore/KC117644) | [KU697330](http://www.ncbi.nlm.nih.gov/nuccore/KU697330) | [KF009695](http://www.ncbi.nlm.nih.gov/nuccore/KF009695) | [KF014059](http://www.ncbi.nlm.nih.gov/nuccore/KF014059) | [KU716085](http://www.ncbi.nlm.nih.gov/nuccore/KU716085) |
|  | BRIP 57383 | *Musa* cv. *Lady finger* | Australia | [KC117643](http://www.ncbi.nlm.nih.gov/nuccore/KC117643) | [KU697329](http://www.ncbi.nlm.nih.gov/nuccore/KU697329) | [KF009694](http://www.ncbi.nlm.nih.gov/nuccore/KF009694) | [KF014058](http://www.ncbi.nlm.nih.gov/nuccore/KF014058) | [KU716084](http://www.ncbi.nlm.nih.gov/nuccore/KU716084) |
| *P. cordylinophila* | MFLUCC10-0166 ^T^ | *Cordyline fruticosa* | Thailand | [KF170287](http://www.ncbi.nlm.nih.gov/nuccore/KF170287) | [KF206242](http://www.ncbi.nlm.nih.gov/nuccore/KF206242) | [KF289172](http://www.ncbi.nlm.nih.gov/nuccore/KF289172) | [KF289295](http://www.ncbi.nlm.nih.gov/nuccore/KF289295) | [KF289076](http://www.ncbi.nlm.nih.gov/nuccore/KF289076) |
|  | MFLUCC 12-0014 | *Cordyline fruticosa* | Thailand | [KF170288](http://www.ncbi.nlm.nih.gov/nuccore/KF170288) | [KF206228](http://www.ncbi.nlm.nih.gov/nuccore/KF206228) | [KF289171](http://www.ncbi.nlm.nih.gov/nuccore/KF289171) | [KF289301](http://www.ncbi.nlm.nih.gov/nuccore/KF289301) | [KF289075](http://www.ncbi.nlm.nih.gov/nuccore/KF289075) |
| *P. doitungensis* | MFLU 21-0175 ^T^ | *Dasymaschalon obtusipetalum* | Thailand | [OK661033](http://www.ncbi.nlm.nih.gov/nuccore/OK661033) | [OK661034](http://www.ncbi.nlm.nih.gov/nuccore/OK661034) | [OL345581](http://www.ncbi.nlm.nih.gov/nuccore/OL345581) | NA | NA |
| *P. eugeniae* | CBS 445.82 ^T^ | *Eugenia aromatica* | Indonesia | [AY042926](http://www.ncbi.nlm.nih.gov/nuccore/AY042926) | [KF206288](http://www.ncbi.nlm.nih.gov/nuccore/KF206288) | [KF289208](http://www.ncbi.nlm.nih.gov/nuccore/KF289208) | [KF289246](http://www.ncbi.nlm.nih.gov/nuccore/KF289246) | [KF289139](http://www.ncbi.nlm.nih.gov/nuccore/KF289139) |
| *P. fallopiae* | MUCC0113 ^T^ | *Fallopia japonica* | Japan | [AB454307](http://www.ncbi.nlm.nih.gov/nuccore/AB454307) | NA | NA | [AB704228](http://www.ncbi.nlm.nih.gov/nuccore/AB704228) | NA |
| *P. guangdongensis* | CFCC 58144 ^T^ | *Viburnum odoratissimum* | China | [OQ202160](http://www.ncbi.nlm.nih.gov/nuccore/OQ202160) | [OQ202170](http://www.ncbi.nlm.nih.gov/nuccore/OQ202170) | [OQ267758](http://www.ncbi.nlm.nih.gov/nuccore/OQ267758) | [OQ267764](http://www.ncbi.nlm.nih.gov/nuccore/OQ267764) | [OQ267770](http://www.ncbi.nlm.nih.gov/nuccore/OQ267770) |
|  | CFCC 58766 | *Viburnum odoratissimum* | China | [OQ202161](http://www.ncbi.nlm.nih.gov/nuccore/OQ202161) | [OQ202171](http://www.ncbi.nlm.nih.gov/nuccore/OQ202171) | [OQ267759](http://www.ncbi.nlm.nih.gov/nuccore/OQ267759) | [OQ267765](http://www.ncbi.nlm.nih.gov/nuccore/OQ267765) | [OQ267771](http://www.ncbi.nlm.nih.gov/nuccore/OQ267771) |
|  | CFCC 58772 | *Viburnum odoratissimum* | China | [OQ202162](http://www.ncbi.nlm.nih.gov/nuccore/OQ202162) | [OQ202172](http://www.ncbi.nlm.nih.gov/nuccore/OQ202172) | [OQ267760](http://www.ncbi.nlm.nih.gov/nuccore/OQ267760) | [OQ267766](http://www.ncbi.nlm.nih.gov/nuccore/OQ267766) | [OQ267772](http://www.ncbi.nlm.nih.gov/nuccore/OQ267772) |
| *P. ilicis-aquifolii* | CGMCC 3.14358 ^T^ | *Ilex aquifolium* | China | [JN692538](http://www.ncbi.nlm.nih.gov/nuccore/JN692538) | NA | [JN692526](http://www.ncbi.nlm.nih.gov/nuccore/JN692526) | [JN692514](http://www.ncbi.nlm.nih.gov/nuccore/JN692514) | NA |
|  | CGMCC 3.14359 | *Ilex aquifolium* | China | [JN692539](http://www.ncbi.nlm.nih.gov/nuccore/JN692539) | NA | [JN692527](http://www.ncbi.nlm.nih.gov/nuccore/JN692527) | [JN692515](http://www.ncbi.nlm.nih.gov/nuccore/JN692515) | NA |
| *P. maculata* | CPC 18347 ^T^ | *Musa* cv. *Golygoly pot-pot* | Australia | [JQ743570](http://www.ncbi.nlm.nih.gov/nuccore/JQ743570) | NA | [KF009700](http://www.ncbi.nlm.nih.gov/nuccore/KF009700) | [KF014016](http://www.ncbi.nlm.nih.gov/nuccore/KF014016) | NA |
|  | BRIP 46622 | *Musa* cv. *Golygoly pot-pot* | Australia | [JQ743567](http://www.ncbi.nlm.nih.gov/nuccore/JQ743567) | NA | [KF009692](http://www.ncbi.nlm.nih.gov/nuccore/KF009692) | [KF014013](http://www.ncbi.nlm.nih.gov/nuccore/KF014013) | NA |
| *P. mangiferae* | IMI 260576 ^T^ | *Mangifera indica* | India | [JF261459](http://www.ncbi.nlm.nih.gov/nuccore/JF261459) | [KF206222](http://www.ncbi.nlm.nih.gov/nuccore/KF206222) | [JF261501](http://www.ncbi.nlm.nih.gov/nuccore/JF261501) | [JF343641](http://www.ncbi.nlm.nih.gov/nuccore/JF343641) | [JF343748](http://www.ncbi.nlm.nih.gov/nuccore/JF343748) |
| *P. mangifera-indicae* | MFLUCC 10-0029^T^ | *Mangifera indica* | Thailand | [KF170305](http://www.ncbi.nlm.nih.gov/nuccore/KF170305) | [KF206240](http://www.ncbi.nlm.nih.gov/nuccore/KF206240) | [KF289190](http://www.ncbi.nlm.nih.gov/nuccore/KF289190) | [KF289296](http://www.ncbi.nlm.nih.gov/nuccore/KF289296) | [KF289121](http://www.ncbi.nlm.nih.gov/nuccore/KF289121) |
| *P. musaechinensis* | GZAAS 6.1247 | *Musa* sp. | China | [KF955294](http://www.ncbi.nlm.nih.gov/nuccore/KF955294) | NA | [KM816639](http://www.ncbi.nlm.nih.gov/nuccore/KM816639) | [KM816627](http://www.ncbi.nlm.nih.gov/nuccore/KM816627) | [KM816633](http://www.ncbi.nlm.nih.gov/nuccore/KM816633) |
|  | GZAAS 6.1384 | *Musa* sp. | China | [KF955295](http://www.ncbi.nlm.nih.gov/nuccore/KF955295) | NA | [KM816640](http://www.ncbi.nlm.nih.gov/nuccore/KM816640) | [KM816628](http://www.ncbi.nlm.nih.gov/nuccore/KM816628) | [KM816634](http://www.ncbi.nlm.nih.gov/nuccore/KM816634) |
| *P. musarum* | BRIP 57803 | *Musa* sp. | Malaysia | [JX997138](http://www.ncbi.nlm.nih.gov/nuccore/JX997138) | NA | [KF009737](http://www.ncbi.nlm.nih.gov/nuccore/KF009737) | [KF014055](http://www.ncbi.nlm.nih.gov/nuccore/KF014055) | NA |
|  | BRIP 58028 | *Musa* sp. | Australia | [KC988377](http://www.ncbi.nlm.nih.gov/nuccore/KC988377) | NA | [KF009738](http://www.ncbi.nlm.nih.gov/nuccore/KF009738) | [KF014054](http://www.ncbi.nlm.nih.gov/nuccore/KF014054) | NA |
| *P. oblongifoliae* | SAUCC210055 | *Garcinia oblongifolia* | China | [OM248442](http://www.ncbi.nlm.nih.gov/nuccore/OM248442) | [OM232085](http://www.ncbi.nlm.nih.gov/nuccore/OM232085) | [OM273890](http://www.ncbi.nlm.nih.gov/nuccore/OM273890) | [OM273894](http://www.ncbi.nlm.nih.gov/nuccore/OM273894) | [OM273898](http://www.ncbi.nlm.nih.gov/nuccore/OM273898) |
|  | SAUCC210052 ^T^ | *Garcinia oblongifolia* | China | [OM248445](http://www.ncbi.nlm.nih.gov/nuccore/OM248445) | [OM232088](http://www.ncbi.nlm.nih.gov/nuccore/OM232088) | [OM273893](http://www.ncbi.nlm.nih.gov/nuccore/OM273893) | [OM273897](http://www.ncbi.nlm.nih.gov/nuccore/OM273897) | [OM273901](http://www.ncbi.nlm.nih.gov/nuccore/OM273901) |
| *P. paracapitalensis* | CPC 26517 ^T^ | *Citrus floridana* | Italy | [KY855622](http://www.ncbi.nlm.nih.gov/nuccore/KY855622) | [KY855796](http://www.ncbi.nlm.nih.gov/nuccore/KY855796) | [KY855951](http://www.ncbi.nlm.nih.gov/nuccore/KY855951) | [KY855677](http://www.ncbi.nlm.nih.gov/nuccore/KY855677) | [KY855735](http://www.ncbi.nlm.nih.gov/nuccore/KY855735) |
|  | CPC 26518 | *Citrus floridana* | Italy | [KY855623](http://www.ncbi.nlm.nih.gov/nuccore/KY855623) | [KY855797](http://www.ncbi.nlm.nih.gov/nuccore/KY855797) | [KY855952](http://www.ncbi.nlm.nih.gov/nuccore/KY855952) | [KY855678](http://www.ncbi.nlm.nih.gov/nuccore/KY855678) | [KY855736](http://www.ncbi.nlm.nih.gov/nuccore/KY855736) |
| *P. parthenocissi* | CBS 111645 ^T^ | *Parthenocissus quinquefolia* | USA | [EU683672](http://www.ncbi.nlm.nih.gov/nuccore/EU683672) | NA | [JN692530](http://www.ncbi.nlm.nih.gov/nuccore/JN692530) | [JN692518](http://www.ncbi.nlm.nih.gov/nuccore/JN692518) | NA |
| *P. partricuspidatae* | NBRC 9466 ^T^ | *Parthenocissus tricuspidata* | Japan | [KJ847424](http://www.ncbi.nlm.nih.gov/nuccore/KJ847424) | NA | [KJ847446](http://www.ncbi.nlm.nih.gov/nuccore/KJ847446) | [KJ847432](http://www.ncbi.nlm.nih.gov/nuccore/KJ847432) | [KJ847440](http://www.ncbi.nlm.nih.gov/nuccore/KJ847440) |
|  | NBRC 9757 | *Parthenocissus tricuspidata* | Japan | [KJ847425](http://www.ncbi.nlm.nih.gov/nuccore/KJ847425) | NA | [KJ847447](http://www.ncbi.nlm.nih.gov/nuccore/KJ847447) | [KJ847433](http://www.ncbi.nlm.nih.gov/nuccore/KJ847433) | [KJ847441](http://www.ncbi.nlm.nih.gov/nuccore/KJ847441) |
| *P. philoprina* | CBS 587.69 | *Ilex aquifolium* | Spain | [KF154278](http://www.ncbi.nlm.nih.gov/nuccore/KF154278) | [KF206297](http://www.ncbi.nlm.nih.gov/nuccore/KF206297) | [KF289206](http://www.ncbi.nlm.nih.gov/nuccore/KF289206) | [KF289250](http://www.ncbi.nlm.nih.gov/nuccore/KF289250) | [KF289137](http://www.ncbi.nlm.nih.gov/nuccore/KF289137) |
| *P. phoenicis* | CBS 147091 | *Phoenix reclinata* | South Africa | [MW883442](http://www.ncbi.nlm.nih.gov/nuccore/MW883442) | [MW883833](http://www.ncbi.nlm.nih.gov/nuccore/MW883833) | [MW890098](http://www.ncbi.nlm.nih.gov/nuccore/MW890098) | [MW890031](http://www.ncbi.nlm.nih.gov/nuccore/MW890031) | [MW890050](http://www.ncbi.nlm.nih.gov/nuccore/MW890050) |
| *P. pterospermi* | SAUCC210104 ^T^ | *Pterospermum heterophyllum* | China | [OM249954](http://www.ncbi.nlm.nih.gov/nuccore/OM249954) | [OM249956](http://www.ncbi.nlm.nih.gov/nuccore/OM249956) | [OM273902](http://www.ncbi.nlm.nih.gov/nuccore/OM273902) | [OM273904](http://www.ncbi.nlm.nih.gov/nuccore/OM273904) | [OM273906](http://www.ncbi.nlm.nih.gov/nuccore/OM273906) |
|  | SAUCC210106 | *Pterospermumheterophyllum* | China | [OM249955](http://www.ncbi.nlm.nih.gov/nuccore/OM249955) | [OM249957](http://www.ncbi.nlm.nih.gov/nuccore/OM249957) | [OM273903](http://www.ncbi.nlm.nih.gov/nuccore/OM273903) | [OM273905](http://www.ncbi.nlm.nih.gov/nuccore/OM273905) | [OM273907](http://www.ncbi.nlm.nih.gov/nuccore/OM273907) |
| *P. rhizophorae* | NCYUCC 19-0352^T^ | *Rhizophora stylosa* | China | [MT360030](http://www.ncbi.nlm.nih.gov/nuccore/MT360030) | [MT360039](http://www.ncbi.nlm.nih.gov/nuccore/MT360039) | NA | [MT363248](http://www.ncbi.nlm.nih.gov/nuccore/MT363248) | [MT363250](http://www.ncbi.nlm.nih.gov/nuccore/MT363250) |
|  | NCYUCC 19-0358 | *Rhizophora stylosa* | China | [MT360031](http://www.ncbi.nlm.nih.gov/nuccore/MT360031) | [MT360040](http://www.ncbi.nlm.nih.gov/nuccore/MT360040) | NA | [MT363249](http://www.ncbi.nlm.nih.gov/nuccore/MT363249) | [MT363251](http://www.ncbi.nlm.nih.gov/nuccore/MT363251) |
| *P. saprophytica* | SAUCC 1516-2 | *the plant saprophytic leaves* | China | OR551459 | OR686933 | OR621092 | OR621084 | OR704557 |
|  | SAUCC 1516-5 | *the plant saprophytic leaves* | China | OR551460 | OR686934 | OR621093 | OR621085 | OR704558 |
| *P. schimae* | CGMCC 3.14354 ^T^ | *Schima superba* | China | [JN692534](http://www.ncbi.nlm.nih.gov/nuccore/JN692534) | NA | [JN692522](http://www.ncbi.nlm.nih.gov/nuccore/JN692522) | [JN692510](http://www.ncbi.nlm.nih.gov/nuccore/JN692510) | [JN692506](http://www.ncbi.nlm.nih.gov/nuccore/JN692506) |
| *P. schimicola* | CGMCC 3.17319 ^T^ | *Schima superba* | China | [KJ847426](http://www.ncbi.nlm.nih.gov/nuccore/KJ847426) | NA | [KJ847448](http://www.ncbi.nlm.nih.gov/nuccore/KJ847448) | [KJ847434](http://www.ncbi.nlm.nih.gov/nuccore/KJ847434) | [KJ854895](http://www.ncbi.nlm.nih.gov/nuccore/KJ854895) |
|  | CGMCC 3.17320 | *Schima superba* | China | [KJ847427](http://www.ncbi.nlm.nih.gov/nuccore/KJ847427) | NA | [KJ847449](http://www.ncbi.nlm.nih.gov/nuccore/KJ847449) | [KJ847435](http://www.ncbi.nlm.nih.gov/nuccore/KJ847435) | [KJ854896](http://www.ncbi.nlm.nih.gov/nuccore/KJ854896) |
| *P. styracicola* | CGMCC3.14985 ^T^ | *Styrax grandiflorus* | China | [JX025040](http://www.ncbi.nlm.nih.gov/nuccore/JX025040) | NA | [JX025045](http://www.ncbi.nlm.nih.gov/nuccore/JX025045) | [JX025035](http://www.ncbi.nlm.nih.gov/nuccore/JX025035) | [JX025030](http://www.ncbi.nlm.nih.gov/nuccore/JX025030) |
|  | CGMCC3.14989 | *Styrax grandiflorus* | China | [JX025041](http://www.ncbi.nlm.nih.gov/nuccore/JX025041) | NA | [JX025046](http://www.ncbi.nlm.nih.gov/nuccore/JX025046) | [JX025036](http://www.ncbi.nlm.nih.gov/nuccore/JX025036) | [JX025031](http://www.ncbi.nlm.nih.gov/nuccore/JX025031) |
| *P. vitis-rotundifoliae* | CGMCC 3.17322 ^T^ | *Vitis rotundifolia* | USA | [KJ847428](http://www.ncbi.nlm.nih.gov/nuccore/KJ847428) | NA | [KJ847450](http://www.ncbi.nlm.nih.gov/nuccore/KJ847450) | [KJ847436](http://www.ncbi.nlm.nih.gov/nuccore/KJ847436) | [KJ847442](http://www.ncbi.nlm.nih.gov/nuccore/KJ847442) |
|  | CGMCC 3.17321 | *Vitis rotundifolia* | USA | [KJ847429](http://www.ncbi.nlm.nih.gov/nuccore/KJ847429) | NA | [KJ847451](http://www.ncbi.nlm.nih.gov/nuccore/KJ847451) | [KJ847437](http://www.ncbi.nlm.nih.gov/nuccore/KJ847437) | [KJ847443](http://www.ncbi.nlm.nih.gov/nuccore/KJ847443) |
| ***Phyllosticta concentrica* species complex** | | | | | | | | |
| *P. anhuiensis* | CFCC 54840^T^ | *Quercus aliena* | China | [OQ202157](http://www.ncbi.nlm.nih.gov/nuccore/OQ202157) | [OQ202167](http://www.ncbi.nlm.nih.gov/nuccore/OQ202167) | [OQ267761](http://www.ncbi.nlm.nih.gov/nuccore/OQ267761) | [OQ267767](http://www.ncbi.nlm.nih.gov/nuccore/OQ267767) | [OQ267773](http://www.ncbi.nlm.nih.gov/nuccore/OQ267773) |
|  | CFCC 55887 | *Quercus aliena* | China | [OQ202158](http://www.ncbi.nlm.nih.gov/nuccore/OQ202158) | [OQ202168](http://www.ncbi.nlm.nih.gov/nuccore/OQ202168) | [OQ267762](http://www.ncbi.nlm.nih.gov/nuccore/OQ267762) | [OQ267768](http://www.ncbi.nlm.nih.gov/nuccore/OQ267768) | [OQ267774](http://www.ncbi.nlm.nih.gov/nuccore/OQ267774) |
|  | CFCC 58849 | *Quercus aliena* | China | [OQ202159](http://www.ncbi.nlm.nih.gov/nuccore/OQ202159) | [OQ202169](http://www.ncbi.nlm.nih.gov/nuccore/OQ202169) | [OQ267763](http://www.ncbi.nlm.nih.gov/nuccore/OQ267763) | [OQ267769](http://www.ncbi.nlm.nih.gov/nuccore/OQ267769) | [OQ267775](http://www.ncbi.nlm.nih.gov/nuccore/OQ267775) |
| *P. aspidistricola* | NBRC 102244 ^T^ | *Aspidistra elatior* | Japan | [AB454314](http://www.ncbi.nlm.nih.gov/nuccore/AB454314) | NA | NA | [AB704204](http://www.ncbi.nlm.nih.gov/nuccore/AB704204) | NA |
| *P. aucubae-japonicae* | MAFF 236703 ^T^ | *Aucuba japonica* | Japan | [KR233300](http://www.ncbi.nlm.nih.gov/nuccore/KR233300) | NA | [KR233310](http://www.ncbi.nlm.nih.gov/nuccore/KR233310) | [KR233305](http://www.ncbi.nlm.nih.gov/nuccore/KR233305) | NA |
| *P. bifrenariae* | CBS 128855 ^T^ | *Bifrenaria harrisoniae* | Brazil | [JF343565](http://www.ncbi.nlm.nih.gov/nuccore/JF343565) | [KF206209](http://www.ncbi.nlm.nih.gov/nuccore/KF206209) | [JF343586](http://www.ncbi.nlm.nih.gov/nuccore/JF343586) | [JF343649](http://www.ncbi.nlm.nih.gov/nuccore/JF343649) | [JF343744](http://www.ncbi.nlm.nih.gov/nuccore/JF343744) |
|  | CPC 17467 | *Bifrenaria harrisoniae* | Brazil | [KF170299](http://www.ncbi.nlm.nih.gov/nuccore/KF170299) | [KF206260](http://www.ncbi.nlm.nih.gov/nuccore/KF206260) | [KF289207](http://www.ncbi.nlm.nih.gov/nuccore/KF289207) | [KF289283](http://www.ncbi.nlm.nih.gov/nuccore/KF289283) | [KF289138](http://www.ncbi.nlm.nih.gov/nuccore/KF289138) |
| *P. catimbauensis* | URM 7672 ^T^ | *Mandevilla catimbauensis* | Brazil | [MF466160](http://www.ncbi.nlm.nih.gov/nuccore/MF466160) | [MF466163](http://www.ncbi.nlm.nih.gov/nuccore/MF466163) | [MF466155](http://www.ncbi.nlm.nih.gov/nuccore/MF466155) | [MF466157](http://www.ncbi.nlm.nih.gov/nuccore/MF466157) | NA |
|  | URM 7674 | *Mandevilla catimbauensis* | Brazil | [MF466161](http://www.ncbi.nlm.nih.gov/nuccore/MF466161) | [MF466164](http://www.ncbi.nlm.nih.gov/nuccore/MF466164) | [MF466153](http://www.ncbi.nlm.nih.gov/nuccore/MF466153) | [MF466158](http://www.ncbi.nlm.nih.gov/nuccore/MF466158) | NA |
| *P. citriasiana* | CBS 120486 ^T^ | *Citrus maxima* | Thailand | [FJ538360](http://www.ncbi.nlm.nih.gov/nuccore/FJ538360) | [KF206314](http://www.ncbi.nlm.nih.gov/nuccore/KF206314) | [FJ538418](http://www.ncbi.nlm.nih.gov/nuccore/FJ538418) | [FJ538476](http://www.ncbi.nlm.nih.gov/nuccore/FJ538476) | [JF343686](http://www.ncbi.nlm.nih.gov/nuccore/JF343686) |
|  | CBS 120487 | *Citrus maxima* | China | [FJ538361](http://www.ncbi.nlm.nih.gov/nuccore/FJ538361) | [KF206313](http://www.ncbi.nlm.nih.gov/nuccore/KF206313) | [FJ538419](http://www.ncbi.nlm.nih.gov/nuccore/FJ538419) | [FJ538477](http://www.ncbi.nlm.nih.gov/nuccore/FJ538477) | [JF343687](http://www.ncbi.nlm.nih.gov/nuccore/JF343687) |
| *P. citribraziliensis* | CBS 100098 ^T^ | *Citrus limon* | Brazil | [FJ538352](http://www.ncbi.nlm.nih.gov/nuccore/FJ538352) | [KF206221](http://www.ncbi.nlm.nih.gov/nuccore/KF206221) | [FJ538410](http://www.ncbi.nlm.nih.gov/nuccore/FJ538410) | [FJ538468](http://www.ncbi.nlm.nih.gov/nuccore/FJ538468) | [JF343691](http://www.ncbi.nlm.nih.gov/nuccore/JF343691) |
| *P. citricarpa* | CBS 127454 ^T^ | *Citrus limon* | Australia | [JF343583](http://www.ncbi.nlm.nih.gov/nuccore/JF343583) | [KF206306](http://www.ncbi.nlm.nih.gov/nuccore/KF206306) | [JF343604](http://www.ncbi.nlm.nih.gov/nuccore/JF343604) | [JF343667](http://www.ncbi.nlm.nih.gov/nuccore/JF343667) | [JF343771](http://www.ncbi.nlm.nih.gov/nuccore/JF343771) |
| *P. citrichinensis* | ZJUCC 200956 ^T^ | *Citrus reticulata* | China | [JN791620](http://www.ncbi.nlm.nih.gov/nuccore/JN791620) | NA | [JN791459](http://www.ncbi.nlm.nih.gov/nuccore/JN791459) | [JN791533](http://www.ncbi.nlm.nih.gov/nuccore/JN791533) | NA |
|  | ZJUCC 2010150 | *Citrus maxima* | China | [JN791662](http://www.ncbi.nlm.nih.gov/nuccore/JN791662) | NA | [JN791514](http://www.ncbi.nlm.nih.gov/nuccore/JN791514) | [JN791582](http://www.ncbi.nlm.nih.gov/nuccore/JN791582) | NA |
| *P. citrimaxima* | MFLUCC 10-0137 ^T^ | *Citrus maxima* | Thailand | [KF170304](http://www.ncbi.nlm.nih.gov/nuccore/KF170304) | [KF206229](http://www.ncbi.nlm.nih.gov/nuccore/KF206229) | [KF289222](http://www.ncbi.nlm.nih.gov/nuccore/KF289222) | [KF289300](http://www.ncbi.nlm.nih.gov/nuccore/KF289300) | [KF289157](http://www.ncbi.nlm.nih.gov/nuccore/KF289157) |
| *P. concentrica* | CBS 937.70 | *Hedera helix* | Italy | [FJ538350](http://www.ncbi.nlm.nih.gov/nuccore/FJ538350) | [KF206291](http://www.ncbi.nlm.nih.gov/nuccore/KF206291) | [FJ538408](http://www.ncbi.nlm.nih.gov/nuccore/FJ538408) | [KF289257](http://www.ncbi.nlm.nih.gov/nuccore/KF289257) | [JF411745](http://www.ncbi.nlm.nih.gov/nuccore/JF411745) |
|  | CPC 18842 ^T^ | *Hedera* sp. | Italy | [KF170310](http://www.ncbi.nlm.nih.gov/nuccore/KF170310) | [KF206256](http://www.ncbi.nlm.nih.gov/nuccore/KF206256) | [KF289228](http://www.ncbi.nlm.nih.gov/nuccore/KF289228) | [KF289288](http://www.ncbi.nlm.nih.gov/nuccore/KF289288) | [KF289163](http://www.ncbi.nlm.nih.gov/nuccore/KF289163) |
| *P. cussonia* | CPC 14873 ^T^ | *Cussonia* sp. | South Africa | [JF343578](http://www.ncbi.nlm.nih.gov/nuccore/JF343578) | [KF206279](http://www.ncbi.nlm.nih.gov/nuccore/KF206279) | [JF343599](http://www.ncbi.nlm.nih.gov/nuccore/JF343599) | [JF343662](http://www.ncbi.nlm.nih.gov/nuccore/JF343662) | [JF343764](http://www.ncbi.nlm.nih.gov/nuccore/JF343764) |
|  | CPC 14875 | *Cussonia* sp. | South Africa | [JF343579](http://www.ncbi.nlm.nih.gov/nuccore/JF343579) | [KF206278](http://www.ncbi.nlm.nih.gov/nuccore/KF206278) | [JF343600](http://www.ncbi.nlm.nih.gov/nuccore/JF343600) | [JF343663](http://www.ncbi.nlm.nih.gov/nuccore/JF343663) | [JF343765](http://www.ncbi.nlm.nih.gov/nuccore/JF343765) |
| ***P. elliptica*** | **CGMCC3.28672^T^** | ***Rubus ellipticus* var. *obcordatus*** | **China** | **PQ891858** | **PQ891850** | **PQ885494** | **PQ885486** | **PQ885502** |
|  | **SAUCC8331-2** | **dead leaves** | **China** | **PQ891859** | **PQ891851** | **PQ885495** | **PQ885487** | **PQ885503** |
| *P. elongata* | CBS 126.22 ^T^ | *Oxycoccus macrocarpos* | USA | [FJ538353](http://www.ncbi.nlm.nih.gov/nuccore/FJ538353) | NA | [FJ538411](http://www.ncbi.nlm.nih.gov/nuccore/FJ538411) | [FJ538469](http://www.ncbi.nlm.nih.gov/nuccore/FJ538469) | [KF289164](http://www.ncbi.nlm.nih.gov/nuccore/KF289164) |
| *P. ericarum* | CBS 132534 ^T^ | *Erica gracilis* | South Africa | [KF206170](http://www.ncbi.nlm.nih.gov/nuccore/KF206170) | [KF206253](http://www.ncbi.nlm.nih.gov/nuccore/KF206253) | [KF289227](http://www.ncbi.nlm.nih.gov/nuccore/KF289227) | [KF289291](http://www.ncbi.nlm.nih.gov/nuccore/KF289291) | [KF289162](http://www.ncbi.nlm.nih.gov/nuccore/KF289162) |
| *P fujianensis* | SAUCC 1366-3 ^T^ | *Lonicera japonica* | China | OR551457 | OR686935 | OR621090 | OR621086 | OR704555 |
|  | SAUCC 1366-5 | *Lonicera japonica* | China | OR551458 | OR686936 | OR621091 | OR621087 | OR704556 |
| *P. gardeniicola* | MUCC0117 | *Gardenia jasminoides* | Japan | [AB454310](http://www.ncbi.nlm.nih.gov/nuccore/AB454310) | NA | NA | [AB704230](http://www.ncbi.nlm.nih.gov/nuccore/AB704230) | NA |
|  | MUCC0089 | *Gardenia jasminoides* | Japan | [AB454303](http://www.ncbi.nlm.nih.gov/nuccore/AB454303) | NA | NA | NA | NA |
| *P. gwangjuensis* | CNUFC NJ1-12 ^T^ | *Torreya nucifera* | Korea | [OK285195](http://www.ncbi.nlm.nih.gov/nuccore/OK285195) | NA | [OM038511](http://www.ncbi.nlm.nih.gov/nuccore/OM038511) | [OM001471](http://www.ncbi.nlm.nih.gov/nuccore/OM001471) | NA |
|  | CNUFC NJ1-12-1 | *Torreya nucifera* | Korea | [OK285196](http://www.ncbi.nlm.nih.gov/nuccore/OK285196) | NA | [OM038512](http://www.ncbi.nlm.nih.gov/nuccore/OM038512) | [OM001472](http://www.ncbi.nlm.nih.gov/nuccore/OM001472) | NA |
| *P. hostae* | CGMCC 3.14355 ^T^ | *Hosta plantaginea* | China | [JN692535](http://www.ncbi.nlm.nih.gov/nuccore/JN692535) | NA | [JN692523](http://www.ncbi.nlm.nih.gov/nuccore/JN692523) | [JN692511](http://www.ncbi.nlm.nih.gov/nuccore/JN692511) | [JN692503](http://www.ncbi.nlm.nih.gov/nuccore/JN692503) |
|  | CGMCC 3.14356 | *Hosta plantaginea* | China | [JN692536](http://www.ncbi.nlm.nih.gov/nuccore/JN692536) | NA | [JN692524](http://www.ncbi.nlm.nih.gov/nuccore/JN692524) | [JN692512](http://www.ncbi.nlm.nih.gov/nuccore/JN692512) | [JN692504](http://www.ncbi.nlm.nih.gov/nuccore/JN692504) |
| *P. hymenocallidicola* | CBS 131309 ^T^ | *Hymenocallis littoralis* | Australia | [JQ044423](http://www.ncbi.nlm.nih.gov/nuccore/JQ044423) | [JQ044443](http://www.ncbi.nlm.nih.gov/nuccore/JQ044443) | [KF289211](http://www.ncbi.nlm.nih.gov/nuccore/KF289211) | [KF289242](http://www.ncbi.nlm.nih.gov/nuccore/KF289242) | [KF289142](http://www.ncbi.nlm.nih.gov/nuccore/KF289142) |
|  | CPC 19331 | *Hymenocallislittoralis* | Australia | [KF170303](http://www.ncbi.nlm.nih.gov/nuccore/KF170303) | [KF206254](http://www.ncbi.nlm.nih.gov/nuccore/KF206254) | [KF289212](http://www.ncbi.nlm.nih.gov/nuccore/KF289212) | [KF289290](http://www.ncbi.nlm.nih.gov/nuccore/KF289290) | [KF289143](http://www.ncbi.nlm.nih.gov/nuccore/KF289143) |
| *P. hypoglossi* | CBS 101.72 | *Ruscus aculeatus* | Italy | [FJ538365](http://www.ncbi.nlm.nih.gov/nuccore/FJ538365) | [KF206326](http://www.ncbi.nlm.nih.gov/nuccore/KF206326) | [FJ538423](http://www.ncbi.nlm.nih.gov/nuccore/FJ538423) | [FJ538481](http://www.ncbi.nlm.nih.gov/nuccore/FJ538481) | [JF343694](http://www.ncbi.nlm.nih.gov/nuccore/JF343694) |
|  | CBS 434.92 ^T^ | *Ruscus aculeatus* | Italy | [FJ538367](http://www.ncbi.nlm.nih.gov/nuccore/FJ538367) | [KF206299](http://www.ncbi.nlm.nih.gov/nuccore/KF206299) | [FJ538425](http://www.ncbi.nlm.nih.gov/nuccore/FJ538425) | [FJ538483](http://www.ncbi.nlm.nih.gov/nuccore/FJ538483) | [JF343695](http://www.ncbi.nlm.nih.gov/nuccore/JF343695) |
| *P. iridigena* | CBS 143410 ^T^ | *Iris* sp. | South Africa | [MG934459](http://www.ncbi.nlm.nih.gov/nuccore/MG934459) | NA | [MG934502](http://www.ncbi.nlm.nih.gov/nuccore/MG934502) | [MG934466](http://www.ncbi.nlm.nih.gov/nuccore/MG934466) | NA |
| *P. kerriae* | MAFF 240047 ^T^ | *Kerria japonica* | Japan | [AB454266](http://www.ncbi.nlm.nih.gov/nuccore/AB454266) | NA | NA | NA | NA |
| *P. kobus* | MUCC0049 | *Magnolia kobus* | Japan | [AB454286](http://www.ncbi.nlm.nih.gov/nuccore/AB454286) | NA | NA | [AB704221](http://www.ncbi.nlm.nih.gov/nuccore/AB704221) | NA |
| *P. ophiopogonis* | KACC 47754 | *Ophiopogon japonicus* | South Korea | [KP197057](http://www.ncbi.nlm.nih.gov/nuccore/KP197057) | NA | NA | NA | NA |
|  | LrLF11 | *Lycoris radiata* | China | [MG543713](http://www.ncbi.nlm.nih.gov/nuccore/MG543713) | NA | NA | NA | NA |
| *P. paracitricarpa* | CPC 27169 ^T^ | *Citrus limon* | Greece | [KY855635](http://www.ncbi.nlm.nih.gov/nuccore/KY855635) | [KY855809](http://www.ncbi.nlm.nih.gov/nuccore/KY855809) | [KY855964](http://www.ncbi.nlm.nih.gov/nuccore/KY855964) | [KY855690](http://www.ncbi.nlm.nih.gov/nuccore/KY855690) | [KY855748](http://www.ncbi.nlm.nih.gov/nuccore/KY855748) |
|  | ZJUCC 200933 | *Citrus sinensis* | China | [JN791626](http://www.ncbi.nlm.nih.gov/nuccore/JN791626) | [KY855813](http://www.ncbi.nlm.nih.gov/nuccore/KY855813) | [JN791468](http://www.ncbi.nlm.nih.gov/nuccore/JN791468) | [JN791544](http://www.ncbi.nlm.nih.gov/nuccore/JN791544) | [KY855752](http://www.ncbi.nlm.nih.gov/nuccore/KY855752) |
| *P.* *pilospora* | MUCC 2912a | *Chamaecyparis pisifera* var. *plumose* | Japan | [LC542597](http://www.ncbi.nlm.nih.gov/nuccore/LC542597) | [LC543423](http://www.ncbi.nlm.nih.gov/nuccore/LC543423) | [LC543445](http://www.ncbi.nlm.nih.gov/nuccore/LC543445) | [LC543465](http://www.ncbi.nlm.nih.gov/nuccore/LC543465) | NA |
| ***P. rhododendri*** | **CGMCC3.28673 ^T^** | ***Rhododendron pulchrum*** | **China** | **PQ891856** | **PQ891848** | **PQ885492** | **PQ885484** | **PQ885500** |
|  | **SAUCC8346-1** | ***Rhododendron pulchrum*** | **China** | **PQ891857** | **PQ891849** | **PQ885493** | **PQ885485** | **PQ885501** |
| *P. speewahensis* | BRIP 58044 ^T^ | Orchids | Australia | [KF017269](http://www.ncbi.nlm.nih.gov/nuccore/KF017269) | NA | [KF017268](http://www.ncbi.nlm.nih.gov/nuccore/KF017268) | NA | NA |
| *P. spinarum* | CBS 292.90 | *Chamaecyparis pisifera* | France | [JF343585](http://www.ncbi.nlm.nih.gov/nuccore/JF343585) | [KF206301](http://www.ncbi.nlm.nih.gov/nuccore/KF206301) | [JF343606](http://www.ncbi.nlm.nih.gov/nuccore/JF343606) | [JF343669](http://www.ncbi.nlm.nih.gov/nuccore/JF343669) | [JF343773](http://www.ncbi.nlm.nih.gov/nuccore/JF343773) |
| *P. turpiniae* | SAUCC2864-3 | *Turpinia arguta* | China | OR551461 | OR686931 | OR621088 | OR704559 | OR704553 |
|  | SAUCC2864-5 | *Turpinia arguta* | China | OR551462 | OR686932 | OR621089 | OR704560 | OR704554 |
| *P. westeae* | BRIP 72390c ^T^ | *Clerodendrum inerme* | Australia | [OP599631](http://www.ncbi.nlm.nih.gov/nuccore/OP599631) | NA | [OP627090](http://www.ncbi.nlm.nih.gov/nuccore/OP627090) | NA | NA |
| ***P. wuzhishanensis*** | **CGMCC3.28670 ^T^** | **Saprophytic leaves** | **China** | **PQ891860** | **PQ891852** | **PQ885496** | **PQ885488** | **PQ885504** |
|  | **SAUCC7810-1** | **Saprophytic leaves** | **China** | **PQ891861** | **PQ891853** | **PQ885497** | **PQ885489** | **PQ885505** |
| ***Phyllosticta cruenta* species complex** | | | | | | | | |
| *P. abieticola* | CBS 112067 | *Abies concolor* | Canada | KF170306 | EU754193 | NA | KF289238 | NA |
| *P. cornicola* | CBS 111639 | *Cornus florida* | USA | KF170307 | NA | NA | KF289234 | NA |
| *P. cruenta* | CBS 858.71 | *Polygonatum odoratum* | Czech Republic | KF170307 | NA | MG934501 | MG934465 | MG934474 |
|  | MUCC0206 | *Polygonatum odoratum* var.*pluriflorum* | Japan | [AB454331](http://www.ncbi.nlm.nih.gov/nuccore/AB454331) | NA | NA | [AB704237](http://www.ncbi.nlm.nih.gov/nuccore/AB704237) | NA |
| *P. cryptomeriae* | KACC 48643 | *Juniperus chinensis* var*. sargentii* | Not given | [MK396559](http://www.ncbi.nlm.nih.gov/nuccore/MK396559) | NA | NA | NA | NA |
|  | MUCC0028 | *Cryptomeria japonica* | Japan | [AB454271](http://www.ncbi.nlm.nih.gov/nuccore/AB454271) | NA | NA | [AB704213](http://www.ncbi.nlm.nih.gov/nuccore/AB704213) | NA |
| *P. foliorum* | CBS 447.68 ^T^ | *Taxus baccata* | Netherlands | [KF170309](http://www.ncbi.nlm.nih.gov/nuccore/KF170309) | [KF206287](http://www.ncbi.nlm.nih.gov/nuccore/KF206287) | [KF289201](http://www.ncbi.nlm.nih.gov/nuccore/KF289201) | [KF289247](http://www.ncbi.nlm.nih.gov/nuccore/KF289247) | [KF289132](http://www.ncbi.nlm.nih.gov/nuccore/KF289132) |
| *P. gaultheriae* | CBS 447.70 ^T^ | *Gaultheria humifusa* | USA | [JN692543](http://www.ncbi.nlm.nih.gov/nuccore/JN692543) | [KF206298](http://www.ncbi.nlm.nih.gov/nuccore/KF206298) | [JN692531](http://www.ncbi.nlm.nih.gov/nuccore/JN692531) | [KF289248](http://www.ncbi.nlm.nih.gov/nuccore/KF289248) | [JN692508](http://www.ncbi.nlm.nih.gov/nuccore/JN692508) |
| *P. hakeicola* | CBS 143492 ^T^ | *Hakea* sp. | Australia | [MH107907](http://www.ncbi.nlm.nih.gov/nuccore/MH107907) | [MH107953](http://www.ncbi.nlm.nih.gov/nuccore/MH107953) | [MH108025](http://www.ncbi.nlm.nih.gov/nuccore/MH108025) | [MH107984](http://www.ncbi.nlm.nih.gov/nuccore/MH107984) | [MH107999](http://www.ncbi.nlm.nih.gov/nuccore/MH107999) |
| *P. hamamelidis* | MUCC149 | *Hamamelis japonica* | Japan | [KF170289](http://www.ncbi.nlm.nih.gov/nuccore/KF170289) | NA | NA | [KF289309](http://www.ncbi.nlm.nih.gov/nuccore/KF289309) | NA |
| *P. hubeiensis* | CGMCC 3.14986 ^T^ | *Viburnum odoratissimim* | China | [JX025037](http://www.ncbi.nlm.nih.gov/nuccore/JX025037) | NA | [JX025042](http://www.ncbi.nlm.nih.gov/nuccore/JX025042) | [JX025032](http://www.ncbi.nlm.nih.gov/nuccore/JX025032) | [JX025027](http://www.ncbi.nlm.nih.gov/nuccore/JX025027) |
|  | CGMCC 3.14987 | *Viburnum odoratissimim* | China | [JX025038](http://www.ncbi.nlm.nih.gov/nuccore/JX025038) | NA | [JX025043](http://www.ncbi.nlm.nih.gov/nuccore/JX025043) | [JX025033](http://www.ncbi.nlm.nih.gov/nuccore/JX025033) | [JX025028](http://www.ncbi.nlm.nih.gov/nuccore/JX025028) |
| *P. illicii* | 24-1-1 ^T^ | *Illicium verum* | China | [MF198235](http://www.ncbi.nlm.nih.gov/nuccore/MF198235) | [MF198240](http://www.ncbi.nlm.nih.gov/nuccore/MF198240) | [MF198237](http://www.ncbi.nlm.nih.gov/nuccore/MF198237) | [MF198243](http://www.ncbi.nlm.nih.gov/nuccore/MF198243) | NA |
|  | 16-16-1 | *Illicium verum* | China | [MF198234](http://www.ncbi.nlm.nih.gov/nuccore/MF198234) | [MF198239](http://www.ncbi.nlm.nih.gov/nuccore/MF198239) | [MF198236](http://www.ncbi.nlm.nih.gov/nuccore/MF198236) | [MF198242](http://www.ncbi.nlm.nih.gov/nuccore/MF198242) | NA |
| *P. leucothoicola* | MUCC0553 ^T^ | *Leucothoe catesbaei* | Japan | [AB454370](http://www.ncbi.nlm.nih.gov/nuccore/AB454370) | [AB454370](http://www.ncbi.nlm.nih.gov/nuccore/AB454370) | NA | [KF289310](http://www.ncbi.nlm.nih.gov/nuccore/KF289310) | NA |
| *P. ligustricola* | MUCC0024 ^T^ | *Ligustrum obtusifolium* | Japan | [AB454269](http://www.ncbi.nlm.nih.gov/nuccore/AB454269) | NA | NA | [AB704212](http://www.ncbi.nlm.nih.gov/nuccore/AB704212) | NA |
| *P. minima* | CBS 585.84 ^T^ | *Acer rubrum* | USA | [KF206176](http://www.ncbi.nlm.nih.gov/nuccore/KF206176) | [KF206286](http://www.ncbi.nlm.nih.gov/nuccore/KF206286) | [KF289204](http://www.ncbi.nlm.nih.gov/nuccore/KF289204) | [KF289249](http://www.ncbi.nlm.nih.gov/nuccore/KF289249) | [KF289135](http://www.ncbi.nlm.nih.gov/nuccore/KF289135) |
| *P. neopyrolae* | CPC 21879 ^T^ | *Pyrola asarifolia* | Japan | [AB454318](http://www.ncbi.nlm.nih.gov/nuccore/AB454318) | [AB454318](http://www.ncbi.nlm.nih.gov/nuccore/AB454318) | NA | [AB704233](http://www.ncbi.nlm.nih.gov/nuccore/AB704233) | NA |
| *P. pachysandricola* | MUCC0124 ^T^ | *Pachysandra terminalis* | Japan | [AB454317](http://www.ncbi.nlm.nih.gov/nuccore/AB454317) | [AB454317](http://www.ncbi.nlm.nih.gov/nuccore/AB454317) | NA | [AB704232](http://www.ncbi.nlm.nih.gov/nuccore/AB704232) | NA |
| *P. paxistimae* | CBS 112527 ^T^ | *Paxistima mysinites* | USA | [KF206172](http://www.ncbi.nlm.nih.gov/nuccore/KF206172) | [KF206320](http://www.ncbi.nlm.nih.gov/nuccore/KF206320) | [KF289209](http://www.ncbi.nlm.nih.gov/nuccore/KF289209) | [KF289239](http://www.ncbi.nlm.nih.gov/nuccore/KF289239) | [KF289140](http://www.ncbi.nlm.nih.gov/nuccore/KF289140) |
| *P. podocarpicola* | CBS 728.79 ^T^ | *Podocarpus maki* | USA | [KF206173](http://www.ncbi.nlm.nih.gov/nuccore/KF206173) | [KF206295](http://www.ncbi.nlm.nih.gov/nuccore/KF206295) | [KF289203](http://www.ncbi.nlm.nih.gov/nuccore/KF289203) | [KF289252](http://www.ncbi.nlm.nih.gov/nuccore/KF289252) | [KF289134](http://www.ncbi.nlm.nih.gov/nuccore/KF289134) |
| *P. pyrolae* | IFO 32652 | *Erica carnea* | Not given | [AB041242](http://www.ncbi.nlm.nih.gov/nuccore/AB041242) | NA | NA | NA | NA |
| *P. rubella* | CBS 111635 ^T^ | *Acer rubrum* | USA | [KF206171](http://www.ncbi.nlm.nih.gov/nuccore/KF206171) | [EU754194](http://www.ncbi.nlm.nih.gov/nuccore/EU754194) | [KF289198](http://www.ncbi.nlm.nih.gov/nuccore/KF289198) | [KF289233](http://www.ncbi.nlm.nih.gov/nuccore/KF289233) | [KF289129](http://www.ncbi.nlm.nih.gov/nuccore/KF289129) |
| *P. sphaeropsoidea* | CBS 756.70 | *Aesculus hippocastanum* | Germany | [AY042934](http://www.ncbi.nlm.nih.gov/nuccore/AY042934) | [KF206294](http://www.ncbi.nlm.nih.gov/nuccore/KF206294) | [KF289202](http://www.ncbi.nlm.nih.gov/nuccore/KF289202) | [KF289253](http://www.ncbi.nlm.nih.gov/nuccore/KF289253) | [KF289133](http://www.ncbi.nlm.nih.gov/nuccore/KF289133) |
| *P. telopeae* | CBS 777.97 ^T^ | *Telopea speciosissima* | Tasmania | [KF206205](http://www.ncbi.nlm.nih.gov/nuccore/KF206205) | [KF206285](http://www.ncbi.nlm.nih.gov/nuccore/KF206285) | [KF289210](http://www.ncbi.nlm.nih.gov/nuccore/KF289210) | [KF289255](http://www.ncbi.nlm.nih.gov/nuccore/KF289255) | [KF289141](http://www.ncbi.nlm.nih.gov/nuccore/KF289141) |
| *P. yuccae* | CBS 112065 | *Yucca elephantipes* | USA | [KF206175](http://www.ncbi.nlm.nih.gov/nuccore/KF206175) | NA | NA | [KF289237](http://www.ncbi.nlm.nih.gov/nuccore/KF289237) | NA |
|  | CBS 117136 | *Yucca elephantipes* | New Zealand | [JN692541](http://www.ncbi.nlm.nih.gov/nuccore/JN692541) | [KF766385](http://www.ncbi.nlm.nih.gov/nuccore/KF766385) | [JN692529](http://www.ncbi.nlm.nih.gov/nuccore/JN692529) | [JN692517](http://www.ncbi.nlm.nih.gov/nuccore/JN692517) | [JN692507](http://www.ncbi.nlm.nih.gov/nuccore/JN692507) |
| ***Phyllosticta owaniana* species complex** | | | | | | | | |
| *P. austroafricana* | CBS 144593 ^T^ | leaf spots of unidentified deciduous tree | South Africa | [MK442613](http://www.ncbi.nlm.nih.gov/nuccore/MK442613) | [MK442549](http://www.ncbi.nlm.nih.gov/nuccore/MK442549) | [MK442704](http://www.ncbi.nlm.nih.gov/nuccore/MK442704) | [MK442640](http://www.ncbi.nlm.nih.gov/nuccore/MK442640) | NA |
| *P. carissicola* | CPC 25665 ^T^ | *Carissa macrocarpa* | South Africa | [KT950849](http://www.ncbi.nlm.nih.gov/nuccore/KT950849) | [KT950863](http://www.ncbi.nlm.nih.gov/nuccore/KT950863) | [KT950879](http://www.ncbi.nlm.nih.gov/nuccore/KT950879) | [KT950872](http://www.ncbi.nlm.nih.gov/nuccore/KT950872) | [KT950876](http://www.ncbi.nlm.nih.gov/nuccore/KT950876) |
| *P. hagahagaensis* | CBS 144592 ^T^ | *Carissa bispinosa* | South Africa | [MK442614](http://www.ncbi.nlm.nih.gov/nuccore/MK442614) | [MK442550](http://www.ncbi.nlm.nih.gov/nuccore/MK442550) | [MK442705](http://www.ncbi.nlm.nih.gov/nuccore/MK442705) | [MK442641](http://www.ncbi.nlm.nih.gov/nuccore/MK442641) | [MK442657](http://www.ncbi.nlm.nih.gov/nuccore/MK442657) |
| *P. owaniana* | CBS 776.97 ^T^ | *Brabejum stellatifolium* | South Africa | [FJ538368](http://www.ncbi.nlm.nih.gov/nuccore/FJ538368) | [KF206293](http://www.ncbi.nlm.nih.gov/nuccore/KF206293) | [FJ538426](http://www.ncbi.nlm.nih.gov/nuccore/FJ538426) | [KF289254](http://www.ncbi.nlm.nih.gov/nuccore/KF289254) | [JF343767](http://www.ncbi.nlm.nih.gov/nuccore/JF343767) |
|  | CPC 14901 | *Brabejum stellatifolium* | South Africa | [JF261462](http://www.ncbi.nlm.nih.gov/nuccore/JF261462) | [KF206303](http://www.ncbi.nlm.nih.gov/nuccore/KF206303) | [JF261504](http://www.ncbi.nlm.nih.gov/nuccore/JF261504) | [KF289243](http://www.ncbi.nlm.nih.gov/nuccore/KF289243) | [JF343766](http://www.ncbi.nlm.nih.gov/nuccore/JF343766) |
| *P. podocarpi* | CBS 111646 | *Podocarpus falcatus* | South Africa | [AF312013](http://www.ncbi.nlm.nih.gov/nuccore/AF312013) | [KF206323](http://www.ncbi.nlm.nih.gov/nuccore/KF206323) | [KC357671](http://www.ncbi.nlm.nih.gov/nuccore/KC357671) | [KC357670](http://www.ncbi.nlm.nih.gov/nuccore/KC357670) | [KF289169](http://www.ncbi.nlm.nih.gov/nuccore/KF289169) |
|  | CBS 111647 | *Podocarpus lanceolata* | South Africa | [KF154276](http://www.ncbi.nlm.nih.gov/nuccore/KF154276) | [KF206322](http://www.ncbi.nlm.nih.gov/nuccore/KF206322) | [KF289232](http://www.ncbi.nlm.nih.gov/nuccore/KF289232) | [KF289235](http://www.ncbi.nlm.nih.gov/nuccore/KF289235) | [KF289168](http://www.ncbi.nlm.nih.gov/nuccore/KF289168) |
| *P. pseudotsugae* | CBS 111649 | *Pseudotsuga menziesii* | USA | [KF154277](http://www.ncbi.nlm.nih.gov/nuccore/KF154277) | [KF206321](http://www.ncbi.nlm.nih.gov/nuccore/KF206321) | [KF289231](http://www.ncbi.nlm.nih.gov/nuccore/KF289231) | [KF289236](http://www.ncbi.nlm.nih.gov/nuccore/KF289236) | [KF289167](http://www.ncbi.nlm.nih.gov/nuccore/KF289167) |
| ***Phyllosticta rhodorae* species complex** | | | | | | | | |
| *P. mimusopisicola* | CBS 138899 ^T^ | *Mimusops zeyheri* | South Africa | [KP004447](http://www.ncbi.nlm.nih.gov/nuccore/KP004447) | [MH878626](http://www.ncbi.nlm.nih.gov/nuccore/MH878626) | NA | NA | NA |
| *P. rhodorae* | CBS 901.69 | *Rhododendron* sp*.* | Netherlands | [KF206174](http://www.ncbi.nlm.nih.gov/nuccore/KF206174) | [KF206292](http://www.ncbi.nlm.nih.gov/nuccore/KF206292) | [KF289230](http://www.ncbi.nlm.nih.gov/nuccore/KF289230) | [KF289256](http://www.ncbi.nlm.nih.gov/nuccore/KF289256) | [KF289166](http://www.ncbi.nlm.nih.gov/nuccore/KF289166) |
| ***Phyllosticta vaccinii species complex*** | | | | | | | | |
| *P. vaccinii* | ATCC 46255 ^T^ | *Vaccinium macrocarpon* | China | [KC193585](http://www.ncbi.nlm.nih.gov/nuccore/KC193585) | NA | [KC193582](http://www.ncbi.nlm.nih.gov/nuccore/KC193582) | [KC193580](http://www.ncbi.nlm.nih.gov/nuccore/KC193580) | [KC193583](http://www.ncbi.nlm.nih.gov/nuccore/KC193583) |
|  | LC 2795 | *Vitis macrocarpon* | USA | [KR233323](http://www.ncbi.nlm.nih.gov/nuccore/KR233323) | NA | NA | NA | NA |
| *P. vacciniicola* | CPC 18590 ^T^ | *Vaccinium macrocarpum* | USA | [KF170312](http://www.ncbi.nlm.nih.gov/nuccore/KF170312) | [KF206257](http://www.ncbi.nlm.nih.gov/nuccore/KF206257) | [KF289229](http://www.ncbi.nlm.nih.gov/nuccore/KF289229) | [KF289287](http://www.ncbi.nlm.nih.gov/nuccore/KF289287) | [KF289165](http://www.ncbi.nlm.nih.gov/nuccore/KF289165) |
| **Outgroup** | | | | | | | | |
| *B. obtusa* | CMW 8232 ^T^ | Conifers | South Africa | [AY972105](http://www.ncbi.nlm.nih.gov/nuccore/AY972105) | NA | [DQ280419](http://www.ncbi.nlm.nih.gov/nuccore/DQ280419) | [AY972111](http://www.ncbi.nlm.nih.gov/nuccore/AY972111) | NA |
| *B. stevensii* | CBS 112553 ^T^ | culture from isotype of *Diplodia mutila* | Not given | [AY259093](http://www.ncbi.nlm.nih.gov/nuccore/AY259093) | [AY928049](http://www.ncbi.nlm.nih.gov/nuccore/AY928049) | [AY573219](http://www.ncbi.nlm.nih.gov/nuccore/AY573219) | NA | NA |

Notes: Ex-type or ex-epitype strains are marked with “T” and the species described in this study was marked in bold.
