# Supplementary material for: ﻿Morphological and phylogenetic analysis reveal three new species Phyllosticta (Phyllostictaceae, Botryosphaeriales) in China
Source: MycoKeys. 2025 May 29;118:35–54. doi: 10.3897/mycokeys.118.153609 (PMC12142213; doi:10.3897/mycokeys.118.153609)
Supplement: Supplementary material 3 — Single phylogenetic analysis [file mycokeys-118-035-s003.pdf]

Phylogram of *Phyllosticta* based on ITS sequences.

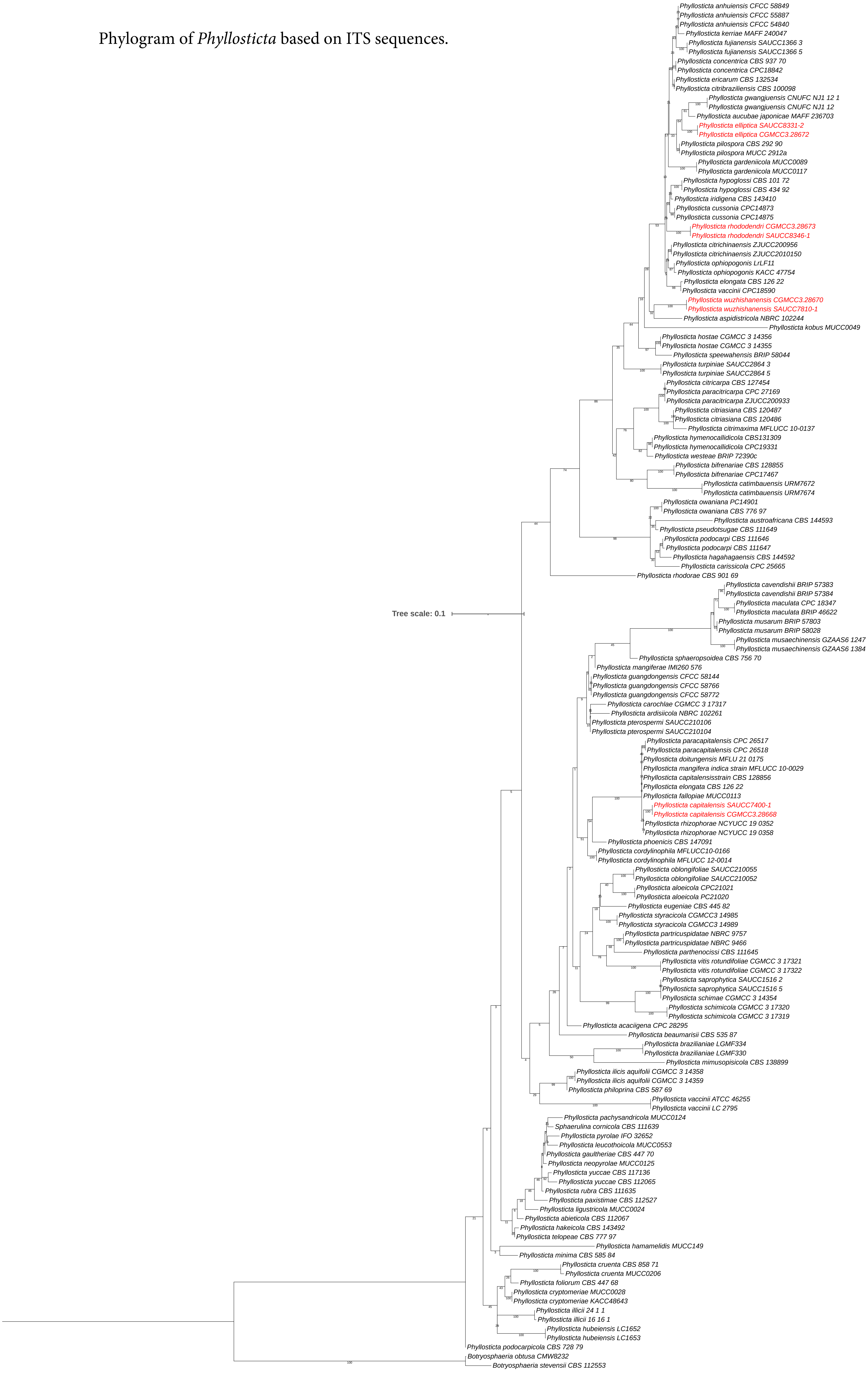

Phylogram of *Phyllosticta* based on LSU sequences.

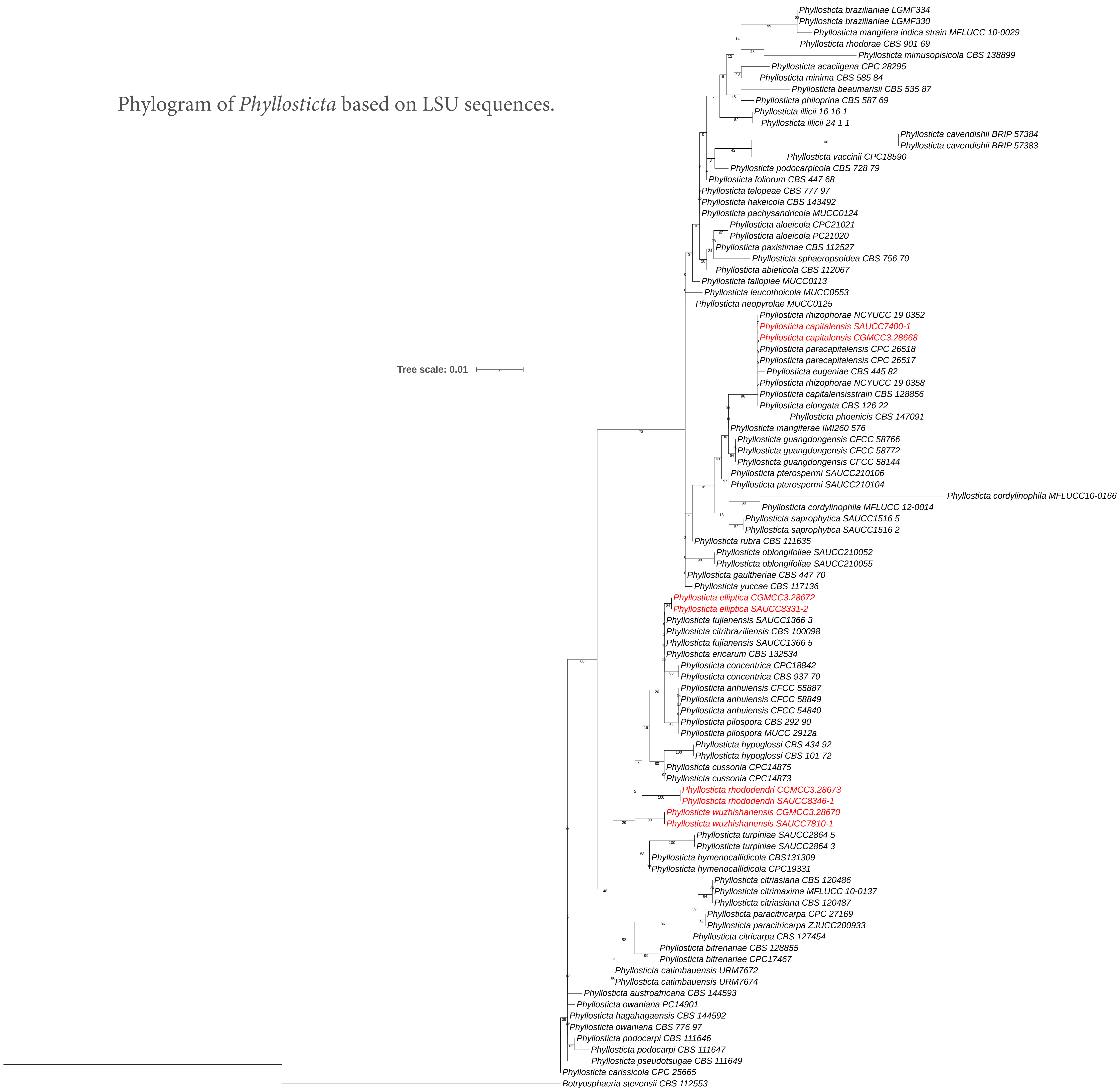

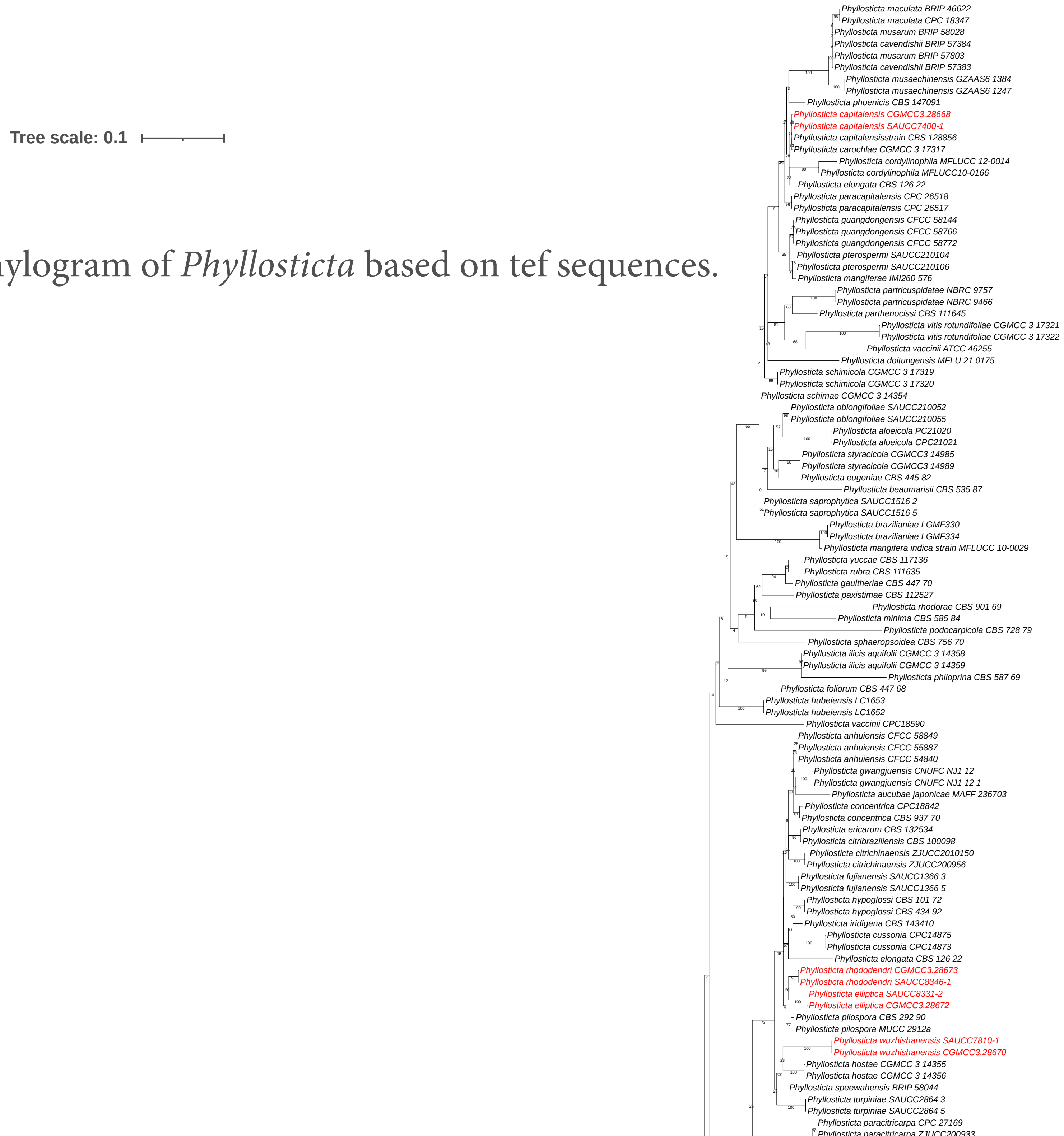

Phylogram of *Phyllosticta* based on tef sequences.

Phylogram of *Phyllosticta* based on act sequences.

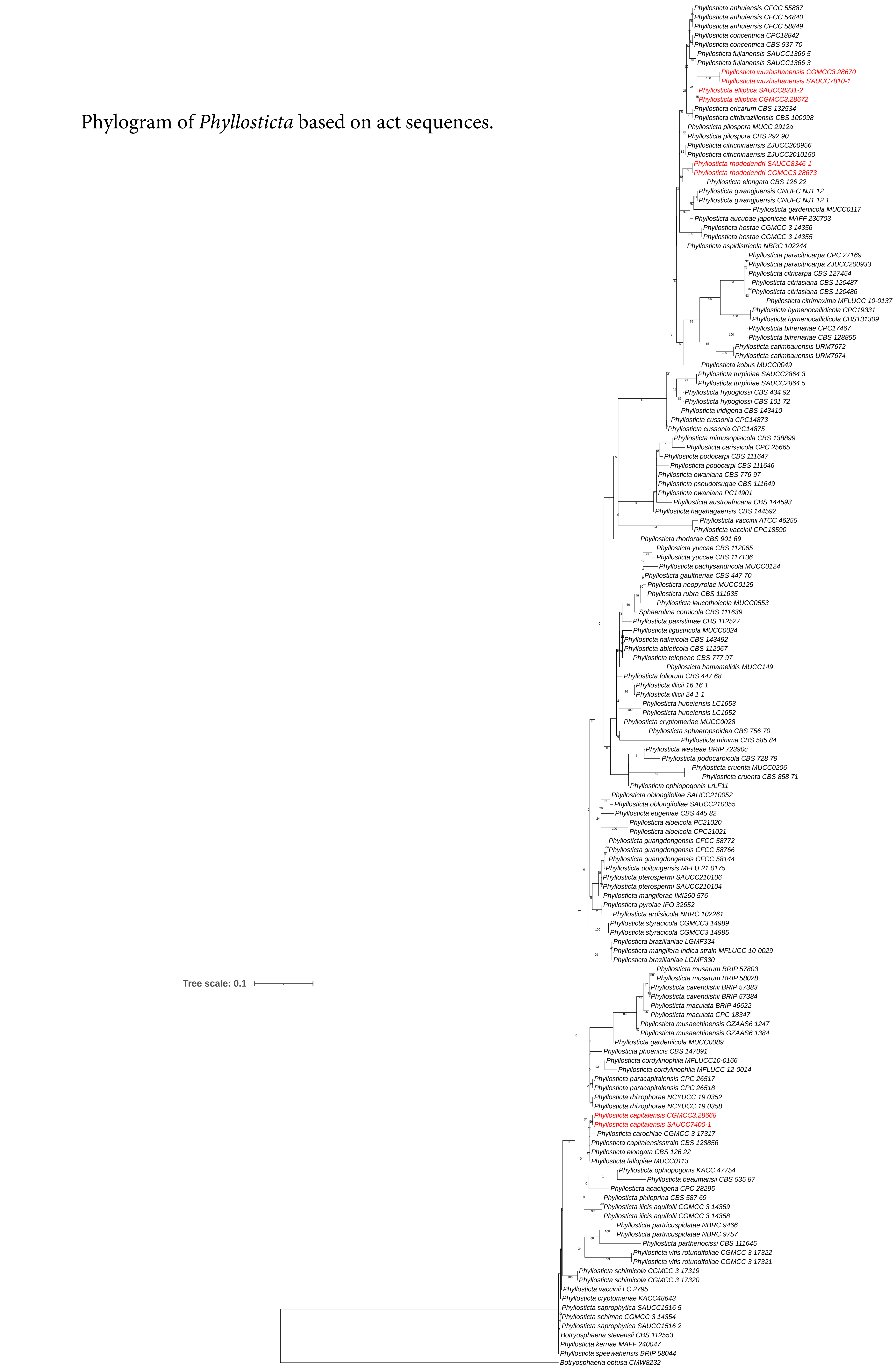

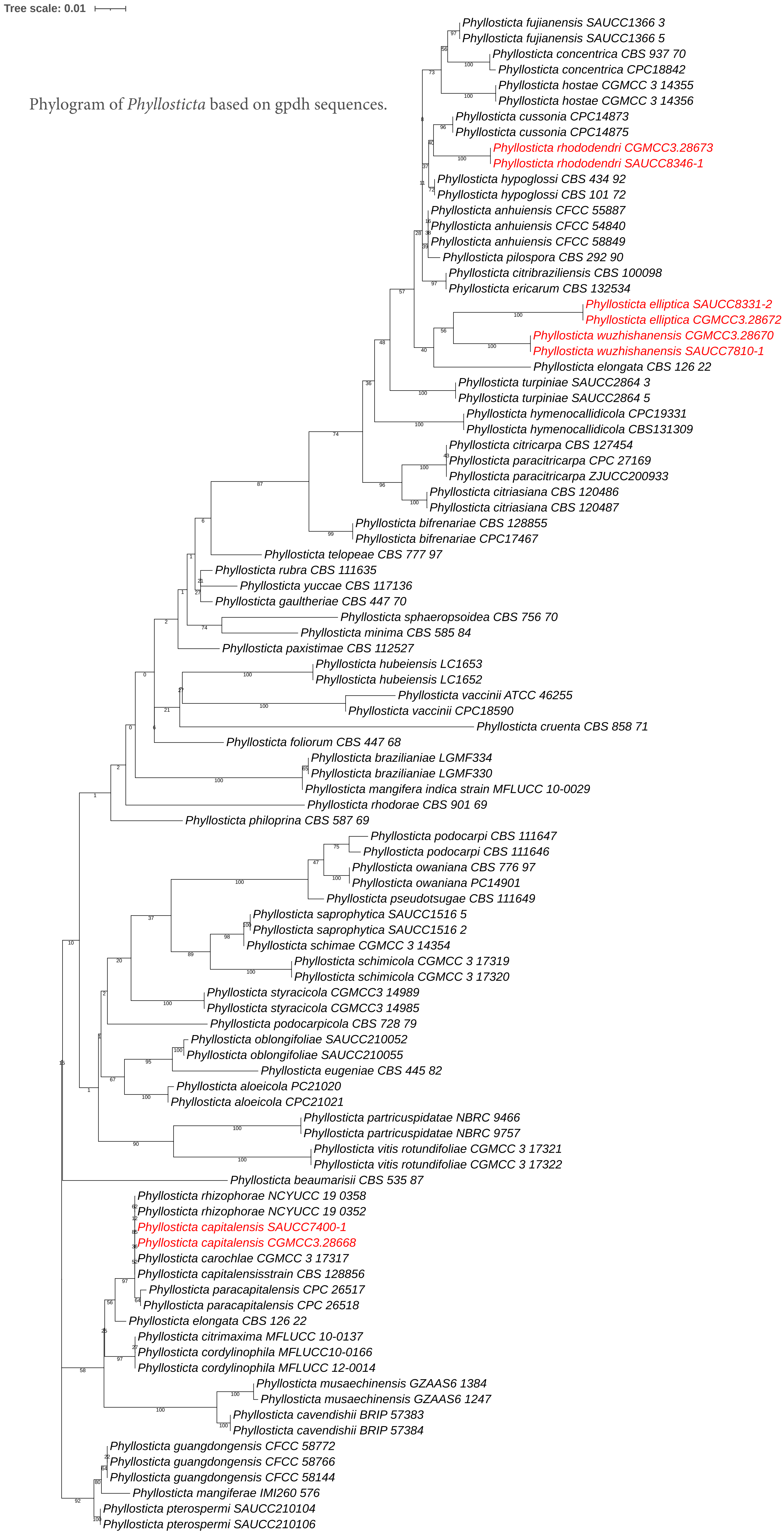

Phylogram of *Phyllosticta* based on gpdh sequences.
